# Insulin and insulin like growth factor II endocytosis and signaling via insulin receptor B

**DOI:** 10.1186/1478-811X-11-18

**Published:** 2013-03-11

**Authors:** Jimena Giudice, Lucia Soledad Barcos, Francisco F Guaimas, Alberto Penas-Steinhardt, Luciana Giordano, Elizabeth A Jares-Erijman, Federico Coluccio Leskow

**Affiliations:** 1 Departamento de Química Biológica, Facultad de Ciencias Exactas y Naturales (FCEN), Universidad de Buenos Aires (UBA), IQUIBICEN, CONICET, Buenos Aires, Argentina; 2 Departamento de Química Orgánica, FCEN, UBA, CIHIDECAR, CONICET, Buenos Aires, Argentina; 3 Instituto de Estudios de la Inmunidad Humoral (IDEHU), CONICET-UBA y Cátedra de Inmunología - Facultad de Farmacia y Bioquímica, UBA, Buenos Aires, Argentina; 4 Laboratory of Cellular Dynamics, Max Planck Institute for Biophysical Chemistry, Göttingen, Germany; 5 Departamento de Ciencias Básicas, Universidad Nacional de Luján, Argentina; 6 Present address: Department of Pathology and Immunology, Baylor College of Medicine, One Baylor Plaza, Houston, TX, 77030, USA

**Keywords:** Insulin / IGF-II, Insulin receptor, Microscopy, Quantum dots, Endocytosis, Signaling

## Abstract

**Background:**

Insulin and insulin-like growth factors (IGFs) act on tetrameric tyrosine kinase receptors controlling essential functions including growth, metabolism, reproduction and longevity. The insulin receptor (IR) binds insulin and IGFs with different affinities triggering different cell responses.

**Results:**

We showed that IGF-II induces cell proliferation and gene transcription when IR-B is over-expressed. We combined biotinylated ligands with streptavidin conjugated quantum dots and visible fluorescent proteins to visualize the binding of IGF-II and insulin to IR-B and their ensuing internalization. By confocal microscopy and flow cytometry in living cells, we studied the internalization kinetic through the IR-B of both IGF-II, known to elicit proliferative responses, and insulin, a regulator of metabolism.

**Conclusions:**

IGF-II promotes a faster internalization of IR-B than insulin. We propose that IGF-II differentially activates mitogenic responses through endosomes, while insulin-activated IR-B remains at the plasma membrane. This fact could facilitate the interaction with key effector molecules involved in metabolism regulation.

## Lay abstract

**Background:** The insulin receptor (IR) responds to insulin and IGF-II regulating glucose metabolism, cellular growth and differentiation. **Results:** Using quantum dot-conjugated IGF-II and insulin we analyzed IR-B endocytosis in correlation with mitogenicity of each ligand. **Conclusions:** IGF-II promotes a fast IR internalization favoring mitogenic signaling whereas insulin-activated IR would endures signaling from the membrane. **Significance:** Ligand-specific redistribution of activated IR ensures signal specificity.

## Background

Insulin and insulin-like growth factors (IGFs) are polypeptide hormones common to all metazoans [1]. They control essential functions including growth, metabolism, reproduction and longevity which are triggered by activation of tetrameric tyrosine kinase receptors [2-9]. During vertebrate and invertebrate embryonic development, insulin and IGFs act as mitogenic growth factors. In postnatal vertebrates, insulin acts as a specialized metabolic hormone regulating glucose homeostasis, whereas IGFs retain their mitogenic functions. Invertebrates possess several insulin-like growth factors genes but only one receptor, DAF-2 [2,10-12]. In contrast, vertebrates express 3 hormone peptides (insulin, IGF-I and IGF-II) and 3 distinct DAF-2 homologous genes: the insulin receptor (*Ir*) [13,14], the IGF-I receptor (*Igf1r*) [15] and the orphan receptor IR related receptor (*Irr*) [16,17]. Moreover, the mammalian *Ir* gene acquired an alternative exon and the ability to regulate its inclusion in a developmental and tissue specific manner. Exon 11 skipping leads to the ancestral IR-A [14], whereas its inclusion leads to the novel IR-B [13]. IR splicing appears altered in aging and in different pathologic states such as type 2 diabetes mellitus and myotonic dystrophy and cancer [18-20].

IGF-I and IGF-II are produced primarily by the liver. IGF-I biosynthesis tightly correlates with the circulating levels of growth hormone, such that *Igf1* gene expression increases 10- to 100-fold between birth and adulthood [21]. Although the biological functions of IGF-I and IGF-II are similar in mammals, in humans circulating IGF-II exceeds IGF-I levels throughout post-natal life. IGFs bind to the IGF-IR, expressed in almost all cell types, promoting cellular growth and proliferation and inhibiting apoptosis. These growth factors stimulate DNA synthesis and regulate development and differentiation in a large variety of cell types, thus playing a key role in the maximum size acquired by an organism. For example, the extreme variability in the size of dogs (the greatest of any vertebrate) is due to polymorphisms present in the *Igf1* gene [22]. It was also shown that a population carrying mutations in the growth hormone receptor gene has lower expression levels of the growth hormone receptor and IGF-I, while exhibiting a very low incidence of cancer and absence of diabetes [23].

The IR and IGF-IR share more than 50% sequence identity and up to 85% in their kinase domain [15,24]. IGF-IR and IR have a conserved modular protein structure [15,25], being able to bind both insulin and IGFs. However, since IGF-IR shows higher affinity for IGF-I and IR for insulin, it is well accepted that insulin metabolic effects are mediated by IR whereas growth stimulation is due to IGF-IR signaling [1].

Almost all mammalian cells express either IR or IGF-IR but at different levels [26-28]. When the two receptors are co-expressed, pro-receptors heterodimerize in the Golgi leading to hybrid receptors IR/IGF-IR [29-33], which presumably would be responsible for the cellular responses to IGF-I, IGF-II and insulin. It has been proposed that hybrid receptors are involved in insulin resistance in type 2 diabetes by lowering the number of hormone binding sites. In addition, a large fraction of hybrid receptors increases the binding sites for IGFs, a situation associated with a number of different types of cancer [27,34].

The above body of information reinforces the importance of understanding the differences between the signaling triggered by different ligands through the axes IR/IGF-IR. The affinities of insulin, IGF-I and IGF-II for IR-A and IR-B as well as for IGF-IR and hybrid receptors have been studied by different groups with contradictory results [27,33,35,36].

In the present study we analyzed the dynamics of signaling and endocytosis of IR-B after stimulation with insulin and IGF-II. We hypothesized that insulin and IGF-II would exhibit different endocytosis dynamics and that these differences would give new insights on the divergence of signaling pathways that could be triggered from the same receptor upon binding of different ligands. We combined techniques that allowed us to study and visualize individual cells by microscopy and flow cytometry. We tracked insulin and IGF-II endocytosis in living cells by using biotinylated ligands conjugated with streptavidin fluorescent nanoparticles (quantum dots or QD). Insulin and IGF-II promoted different endocytosis dynamic of IR-B, suggesting a mechanism for the divergent signaling pathways that can be trigger from a same receptor upon binding different ligands.

## Results

### IGF-II induces IR-B phosphorylation

**Figure 1:**
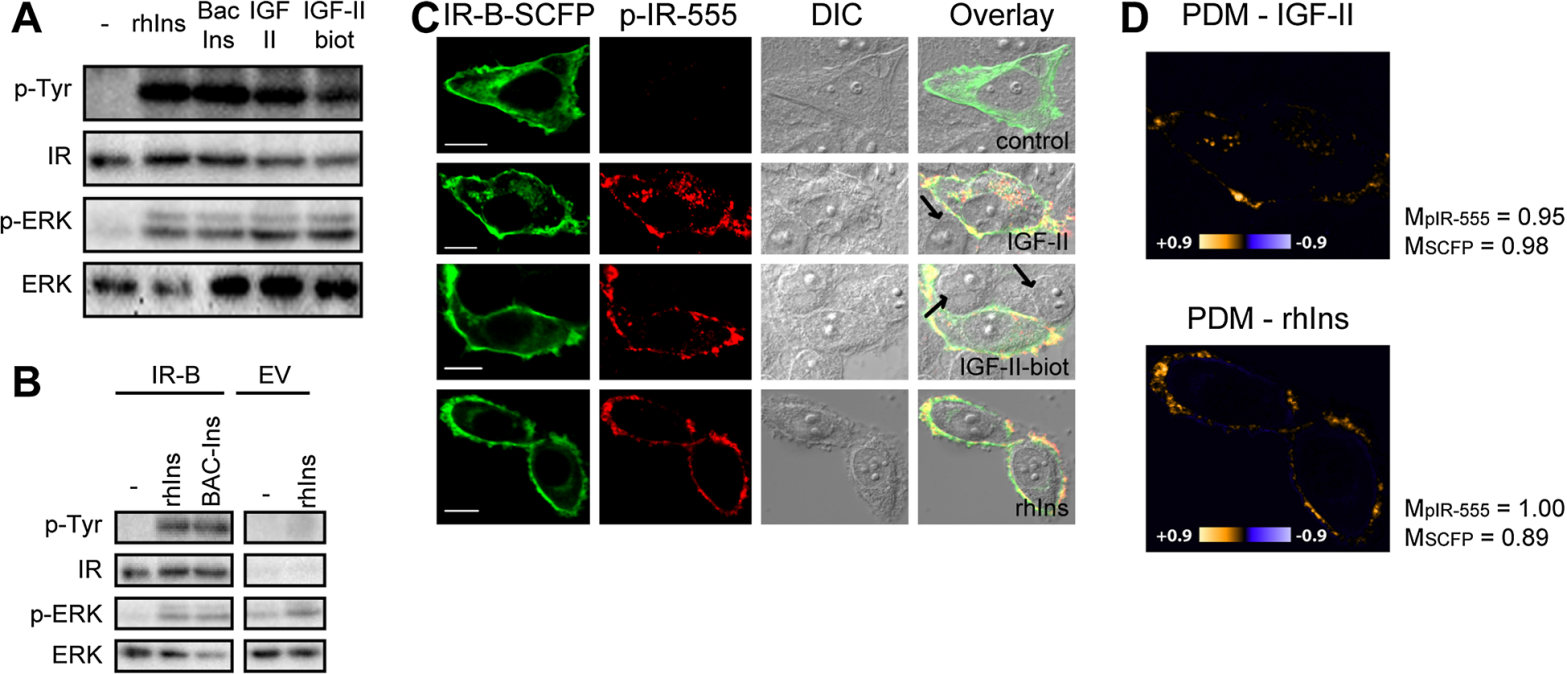
**IR activation by IGF-II and IGF-II-biotin. A**. HeLa cells over-expressing IR-B were starved for 16 hours and stimulated for 5 min with 100 nM rhIns, BAC-Ins, IGF-II or IGF-II-biot at 37°C. Lysates were analyzed by Western blot with anti-phosphorylated-Tyrosine (p-Tyr) and anti-IR-β subunit antibodies; and anti-phosphorylated-ERK 1/2 (p-ERK) and anti-ERK 1/2 antibodies. **B**. HeLa cells were transfected with pcDNA3-IR-B or EV stimulated or not with insulin for 5 min (bands were cut from the same gel). **C**. HeLa cells expressing IR-B-SCFP stimulated with 100 nM IGF-II, IGF-II-biot or rhIns for 5 min at 37°C, fixed in cold methanol and analyzed by immunofluorescence with anti-phosphorylated-IR-β subunit (Tyrosine 1361) antibody and a secondary antibody conjugated with Alexa fluor 555. Imaging was performed by confocal microscopy (Olympus Fluoview FV1000). Scale bars: 10 μm. Arrows indicate that non transfected cells do not show activation signal upon ligand stimulation. Arrows indicate non transfected cells. **D**. Colocalization analysis performed with Image J. Product of the differences from the mean (PDM) plots and Manders coefficients for each channel (M_pIR-555 _and M_SCFP_) are shown for stimulation with IGF-II and rhIns.

We evaluated the effect of IGF-II on IR-B activation by confocal microscopy and Western blot. HeLa cells were transfected with pcDNA3-IR-B, serum starved and stimulated with 100 nM human IGF-II or recombinant human insulin (rhIns) for 5 min at 37°C. Lysates were assayed by Western blot, showing that IR-B responds to IGF-II and to rhIns (Figure 1A). Immunoblotting was carried on using a general antibody against phosphorylated tyrosine (PY20). The activation signal upon ligand binding was only present in cells over-expressing IR-B showing a band of the correct size of the β-subunit of the insulin receptor (~95 kDa). Figure 1B shows a representative Western blot where the cells were transfected with the empty pcDNA3 (EV) showing that the levels of endogenous IR were almost not detected, neither its phosphorylation.

We evaluated HeLa endogenous levels of IR-A and IR-B by reverse transcription and PCR (RT-PCR) observing a 24% ± 6% exon 11 inclusion (Additional file 1: Figure S1A). This is consistent with HeLa’s tumor origin and the reports showing a switch in the ratio of IR-A/IR-B in cancer cells [23,33,37]. Endogenous levels of IR and IGF-IR mRNA were measured by RT-PCR and normalized to GADPH mRNA showing a mRNA relative abundance of 1.1% ± 0.4% for IR and 8.1% ± 1.0% for IGF-IR (Additional file 1: Figure S1B). Correct identity of overexpressed IR-B was analyzed by sequencing and PCR (Additional file 1: Figure S1C).

In order to discriminate the contribution of the transfected IR-B from the endogenous receptors we measured IR activation in single cells expressing IR-B fused with the super cyan fluorescent protein (SCFP) (IR-B-SCFP) by immunofluorescence using a specific antibody against phosphorylated IR-β subunit (Tyrosine 1361). We detected activation of IR-B at the plasma membrane after stimulating with 100 nM IGF-II or rhIns (Figure 1C). Non-transfected cells did not show any activation signal (pointed with arrows in Figure 1C). Signal colocalization between IR-B-SCFP and phosphorylated IR-β confirms that receptor activation triggered by ligand binding is originated from the over-expressed IR-B (Figure 1D).

We next estimated IR transfection efficiency ranging between 30 and 60% by microscopy using DIC (total cells) and fluorescence (transfected) images (*n* = 219 cells for IR fused to visible fluorescent proteins (VFP) and *n* = 308 cells for IR from at least 3 images from 3 independent experiments) (Additional file 2: Figure S2).

### IGF-II induces cell proliferation through IR-B

**Figure 2:**
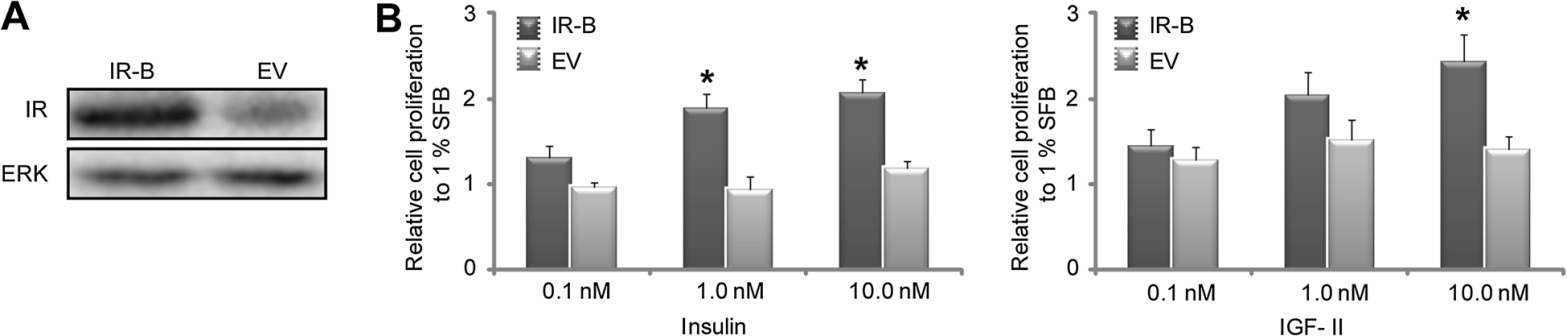
**Proliferative response of IGF-II and rhIns through IR-B. A**. HeLa cells transiently transfected with pcDNA3-IR-B or EV were assayed by Western blot detecting the expression of IR and ERK 1/2 (loading control). **B**. MTS assay in cells transiently transfected with pcDNA3-IR-B or EV stimulated with 0.1 nM, 1.0 nM and 10.0 nM IGF-II or rhIns for 2 days. The results are shown as the mean ± s.e.m (*n* = 3 independent experiments). Asterisks indicate significant differences between IR and EV (*p* < 0.05).

It is known that IGF-II is a mitogenic ligand for the IR [37,38]. In order to evaluate insulin and IGF-II-induced proliferation though IR-B, cells transfected with pcDNA3-IR-B or the EV were starved with 1% FBS overnight and then treated with vehicle or 0.1 nM, 1.0 nM or 10.0 nM IGF-II or rhIns for 2 days after which cell proliferation was measured by a non-radioactive assay. Data was normalized to those obtained with vehicle treated cells. Cells transfected with EV did not show any dependence on ligand concentration (Figure 2). This result suggests negligible contribution of the endogenous levels of IR and IGF-IR in this proliferation assay. In contrast, both insulin and IGF-II induced a proliferative response in a dose dependent manner in IR-B over-expressing cells (Figure 2) (insulin: 1.3 ± 0.1 for 0.1 nM, 1.9 ± 0.2 for 1.0 nM and 2.1 ± 0.1 for 10.0 nM; IGF-II: 1.4 ± 0.2 for 0.1 nM, 2.1 ± 0.3 for 1.0 nM and 2.4 ± 0.3 for 10.0 nM). In both cases these results were statistical different than those obtained in cells transfected with EV.

These data suggest that IGF-II induces cell proliferation via IR-B. Although a higher proliferative response was observed for IGF-II as compare to insulin, the differences between both ligands were not statistically significant.

### IGF-II induces AP-1 dependent gene transcription through IR-B

**Figure 3:**
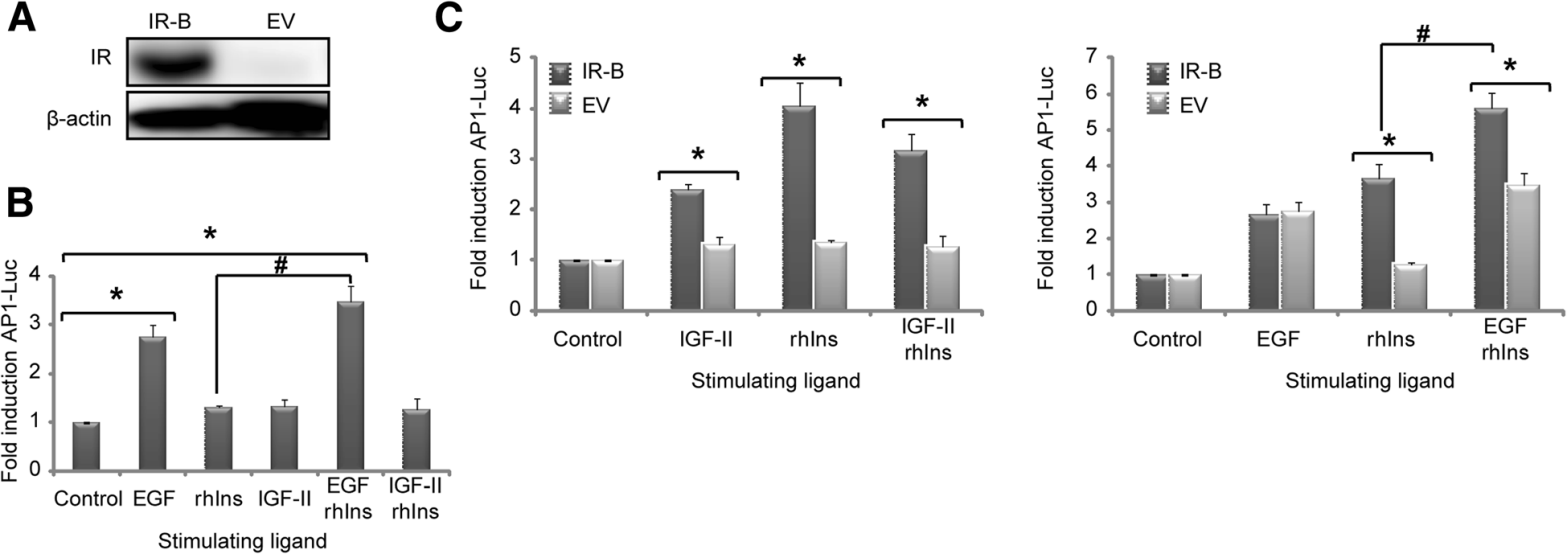
**AP-1 mediated gene transcription induced by IGF-II via IR-B. A**. HeLa cells transiently transfected with pcDNA3-IR-B or the EV assayed by Western blot detecting the expression of IR and β-actin (loading control). **B**. Luciferase activity on cells transfected with pAP1-Luc and EV. After 24 hours, serum was depleted for 1 day and cells were stimulated for 16 hours with: 1.6 nM EGF, 100 nM rhIns, 100 nM IGF-II, 1.6 nM EGF + 100nM rhIns or 100 nM IGF-II + 100 nM rhIns. Luciferase induction was calculated as the ratio between stimulated and non-stimulated cells. The results are shown as the mean ± s.e.m (*n* = 4 independent experiments for EGF, rhIns and EGF + rhIns; *n* = 3 independent experiments for IGF-II and IGF-II + rhIns and *n* = 7 for rhIns). Asterisks indicate significant differences (*p* = 0.005) with non transfected cells (control). **C**. Cells cotransfected with pAP1-Luc and pcDNA3-IR-B or EV were stimulated with: 1.6 nM EGF, 100 nM rhIns, 100 nM IGF-II, 1.6 nM EGF + 100nM rhIns or 100 nM IGF-II + 100 nM rhIns for 16 hours and luciferase activity was measured. The results are shown as the mean ± s.e.m. (*n* = 4 independent experiments for EGF, rhIns and EGF + rhIns; *n* = 3 independent experiments for IGF-II and IGF-II + rhIns). Asterisks indicate significant differences (*p* < 0.01) between cells over-expressing IR-B with cells transfected with EV. In **B** and **C** panels the symbol # indicates that AP-1-Luc response after EGF + rhIns stimulation is significantly different form rhIns alone either in cells over-expressing IR-B (*p* = 0.007) or in cells transfected with EV (*p* = 0.005).

We measured AP-1 dependent gene transcription using a Luciferase reporter assay in cells over-expressing IR-B (Figure 3A). For this purpose, first we evaluated the effects of IGF-II and insulin on endogenous receptors. HeLa cells are derived from an epithelial tumor and are well known to respond to the epidermal growth factor (EGF), that can be suitably used as a positive control (Figure 3B). No effect on gene transcription was observed upon stimulation with IGF-II and/or rhIns. These results confirm an insignificant contribution of endogenous IR and IGF-IR. Next, we evaluated, in IR-B expressing HeLa cells, the effects of EGF in combination with IGF-II or rhIns on AP-1 induction. IR-B over-expressing cells showed a significant response to rhIns and IGF-II, without affecting EGF induction (Figure 3C, right). Co-stimulation with EGF and rhIns showed an additive effect on AP-1 induction, suggesting that both ligands activate AP-1 through different pathways. This response was statistically different from rhIns alone (*p* ≤ 0.005). In contrast, cell*s* treated with rhIns + IGF-II did not exhibit a combined response, indicating that these ligands induced AP-1 through the same receptor (Figure 3C, left).

### Imaging of ligand-receptor complexes endocytosis

In order to visualize the ligand receptor endocytosis using QD, commercially available biotinylated ligands, biotin amido caproyl insulin (BAC-Ins) and IGF-II-biot, were used [39]. We confirmed by Western blot and immunofluorescence that BAC-Ins and IGF-II-biot were capable of activating IR-B (Figure 1A-B). To visualize the receptor we used IR-B fused with different VFPs. These proteins localized properly at the plasma membrane and were positively stained with two different antibodies against regions of the N-terminus of the α-subunit of IR (N20 and H78) (Additional file 3: Figure S3).

**Figure 4:**
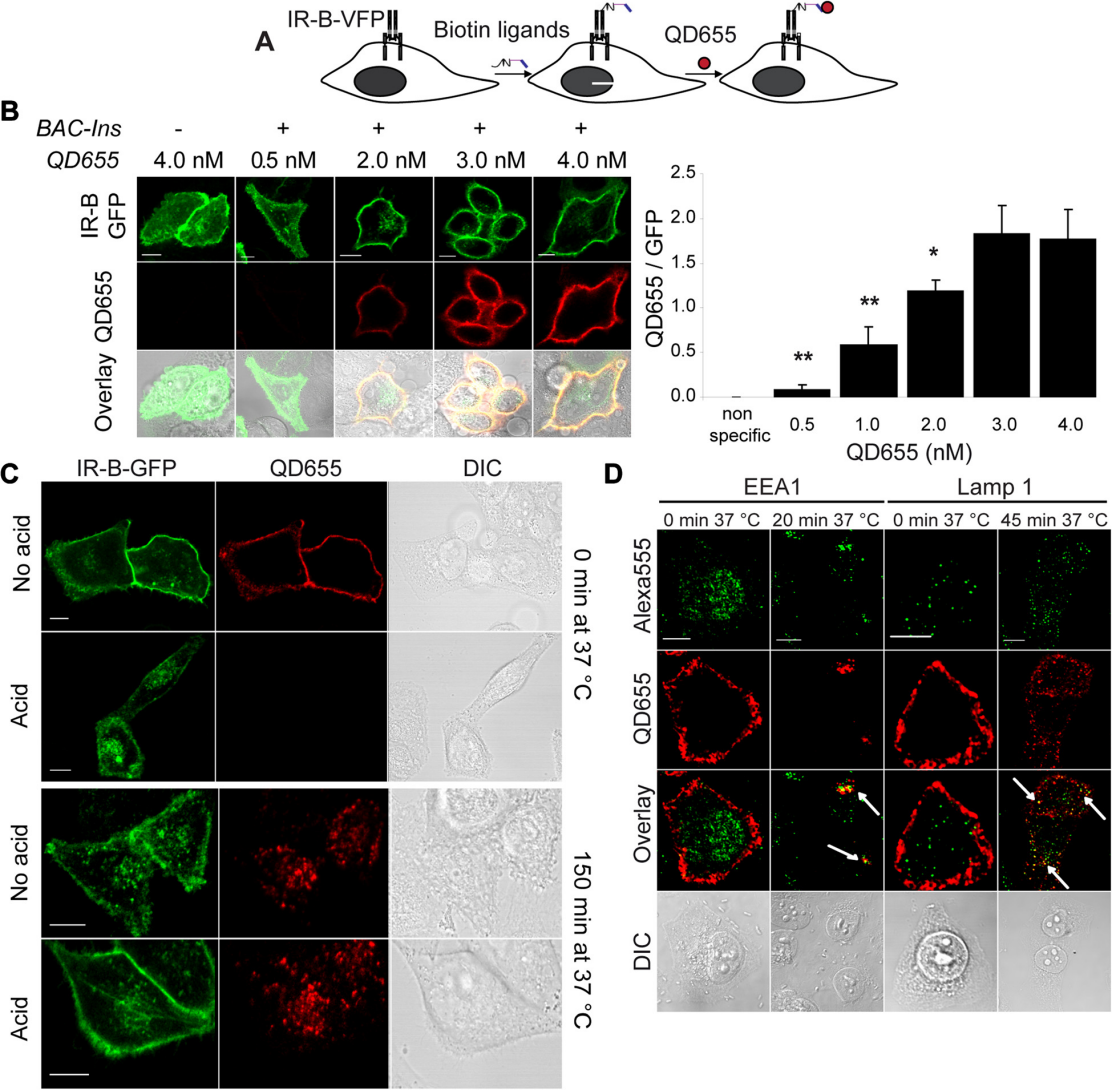
**Imaging of biotinylated ligands binding and internalization by QDs. A. **Labeling strategy. **B. **QD655 concentration curve: HeLa cells expressing IR-B-GFP were labeled with 50 nM BAC-Ins for 15 min and different concentration of QD655 (0.5 nM to 4.0 nM) for 10 min at 15°C. Quantification of the ratio between QD655 and GFP signal (GFP/QD655). Asterisks indicate significant differences: * *p* < 0.0001 and ** *p* = 0.01 (*n* = 6–10 cells). **C**. HeLa cells expressing IR-B-GFP were labeled *in vivo* with 50 nM BAC-Ins and 1 nM QD655 at 15°C and further either directly fixed in 3.7% PFA or treated with acid solution (0.5 M NaCl, 0.1 M Na-glycine pH 3.0) for 5 min and washed before fixation. The two bottom panels show similar experiments but the cells were incubated at 37°C for 150 min before acid treatment and/or fixation. In all the cases imaging was performed by confocal microscopy (Zeiss LSM 510 Meta). Scale bars: 10 μm. **D. **HeLa cells expressing IR-B were labeled with 50 nM BAC-Ins and 1 nM QD655 at room temperature. Samples were incubated or not at 37°C and then fixed in 3.7% PFA. Immunofluorescence was performed with antibodies against early endosomes (EEA1) or CD63 (Lamp 1). Secondary antibodies were conjugated with Alexa fluor 555. Imaging was performed by confocal microscopy (Olympus Fluoview FV 1000). Scale bars: 10 μm.

The two step labeling strategy is schematically summarized in Figure 4A. Briefly, live HeLa cells expressing IR-B were treated at 15°C or room temperature with biotinylated ligands for 15 min. After washing the free ligand, cells were incubated 10 min with streptavidin conjugated QD655 (QD with emission peak at 655 nm), washed to remove unbound QD655 and incubated at 37°C for different time periods.

To evaluate the optimal QD655 concentration, HeLa cells expressing IR-B-GFP were incubated with 50 nM BAC-Ins followed by variable concentrations of QD655 (Figure 4B). Cells were fixed with PFA and imaged by confocal microscopy. Control cells without previous incubation with BAC-Ins did not show any signal (Figure 4B, first column). We calculated the ratio between QD655 and GFP signals (see Methods) observing a dose response curve. These ratios were significantly different (*p* ≤ 0.01). From these results a non saturating concentration of 1 nM QD655 was chosen for further work

The internalization of IR-B-GFP in living cells was analyzed by sequentially labeling HeLa cells with 50 nM BAC-Ins and 1 nM QD655. Cells were directly fixed in 3.7% PFA or incubated at 37°C for 150 min before fixation (Figure 4C). At the beginning GFP and QD655 signals co-localized at the plasma membrane. By contrast, after 150 min at 37°C the majority of QD655 signal was located in the cytoplasm, in the perinuclear region. In order to confirm the internalization, cells were treated with an acid solution (0.5 M NaCl, 0.1 M Na-glycine pH 3.0) for 5 min and washed before fixation. Acid would induce the dissociation between ligand and receptor at the cell surface [40]. These experiments showed that, while in samples that were not incubated at 37°C the acid treatment removed completely QD655 signal (without affecting IR-B-VFP signal), cells incubated at 37°C maintained the internalized QD655 signal. To evaluate whether the size or the nature of the nanoparticles could be affecting the observed results, similar experiments were performed but treating cells with 50 nM BAC-Ins and sequentially with 1 nM fluorescent streptavidin (SA-atto 550) (Additional file 4: Figure S4A). Additionally, cells were directly incubated with 50 nM fluorescently labeled insulin (FITC-insulin) (Additional file 4: Figure S4B). Although brightness and photostability were compromised, both strategies showed a similar labeling pattern as compare to QD655 either at the onset or after internalization.

In order to trace the insulin-IR endocytic pathway we performed immunofluorescence experiments using early endosomes (EEA1) and lysosome markers (Lamp 1). After 20 min, co-localization between QD655 and early endosomes was observed while at 45 min, the QD655 signal was mainly located at perinuclear lysosomes (Figure 4D).

### Visualization of IGF-II bound to IR-B and its endocytosis

**Figure 5:**
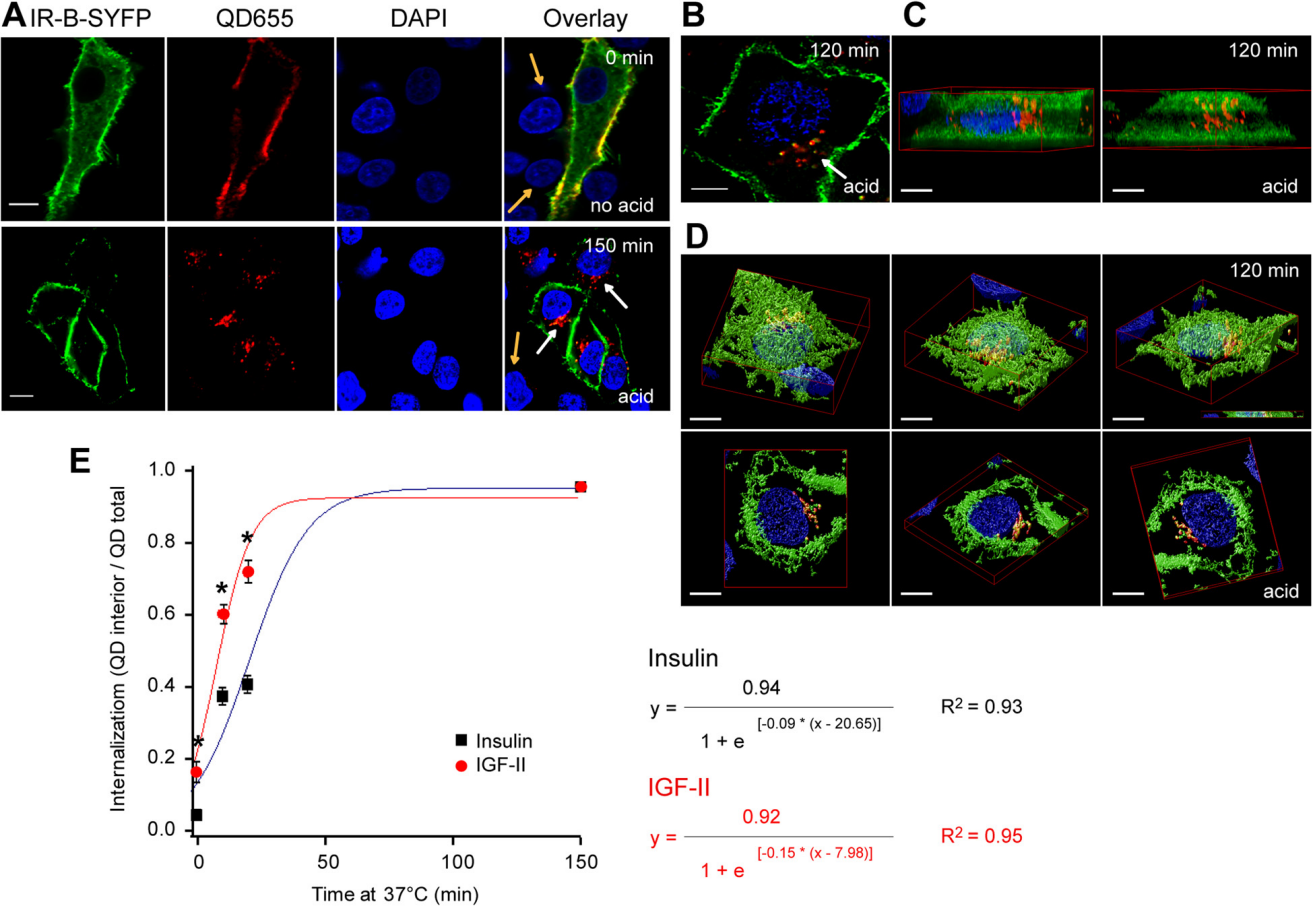
**IGF-II binding and internalization via IR by confocal microscopy. A. **HeLa cells expressing IR-B-SYFP were labeled *in vivo *with 50 nM IGF-II-biot and 2 nM QD655 at room temperature and fixed in 3.7% PFA. Lower panel shows cells incubated at 37°C for 150 min after labeling and treated with acid (0.5 M NaCl, 0.1 M Na-glycine pH 3) for 5 min before fixation. Imaging was performed by confocal microscopy (Olympus Fluoview FV 1000). Scale bars: 10 μm. **B. **HeLa cells expressing IR-B-GFP were labeled *in vivo *with 50 nM IGF-II-biot and 2 nM QD655, incubated for 1 hour at 37°C, then with 0.1 mM Na-glycine pH 3, NaCl 0.5 M for 5 min and fixed in 3.7% PFA. Images were taken with a confocal microscope (Olympus Fluoview FV 1000) by acquisition of 64 z-slices with 16 μm step size. Deconvolution was performed with Huygens Scientific Volume Imaging Huygens Professional Version 3.6, applying the Quick maximum likelihood estimation algorithm. Tridimensional reconstructions are shown. **C.** Maximum intensity projection. **D. **Surface renderer. Different views are shown. Lower panels show views of 14 z-slices section (2.24 μm) that is shown in the right lower corner of the third panel. Scale bars: 5 μm. **E. **Quantification of the degree of internalization of insulin and IGF-II (ratio between QD_interior _and QD_total_). The results are shown as the mean ± s.e.m (*n* = 19–29 cells). Asterisks indicate significant differences between ligands (*p* ≤ 0.02). In **A **and **B **white arrows indicate internalized QD655 and orange arrows label non transfected cells.

We have previously shown that IGF-II and IGF-II-biot induce IR-B activation followed by ERK 1/2 phosphorylation, AP-1 gene transcription and cell proliferation. To study IGF-II induced endocytosis of IR-B in individual cells, HeLa cells expressing IR-B-SYFP were incubated with 50 nM IGF-II-biot for 15 min and labeled with 1 nM QD655 for 10 min at room temperature. Confocal microscopy revealed that IGF-II bound specifically to IR-B-SYFP: no signal was detected in non-transfected cells (Figure 5A, arrows). When cells were incubated at 37°C for 150 min after labeling, internalization of IGF-II-QD655 was detected only in transfected cells. Internalization was confirmed by exposure of the cells to an acidic solution (5 min, 0.5 M NaCl, 0.1 M Na-glycine pH 3.0) before fixation. The data demonstrates that ligand-QD complexes were exclusively localized at the plasma membrane and internalized only after subsequent incubation at 37°C. QD655 signal was only detected inside the cell (Figure 5A, second row panels). The intracellular localization of IGF-II-QD655 complexes was assessed in more detail by optical sectioning. We acquired 64 confocal *z*-slices with a 16 μm step size of cells expressing IR-B-GFP labeled as described above, incubated for 150 min at 37°C and treated for 5 min with acid before fixation. Deconvolution of the images by a Quick Maximum Likelihood Estimation (QMLE) algorithm and tridimensional reconstruction of the cells evidenced the presence of IGF-II-QD655 in the perinuclear region partially co-localizing with IR-B-GFP (Figure 5B-D and Additional file 5: Movie S1).

### Kinetics of IGF-II and insulin internalization by microscopy

The kinetics of ligand internalization of IGF-II and insulin was quantified by labeling HeLa cells expressing IR-B-SYFP with 50 nM IGF-II or BAC-Ins and 1 nM QD655 and further incubating at 37°C for 10, 20 or 150 min. QD655 signal inside the cells increased over time, indicating that endocytosis had taken place (Figure 5A). The extent of endocytosis was quantified after segmentation of each cell into *membrane* and *interior,* calculating the ratio between the signal from the cytosol (QD_interior_) over the total (QD_total_ = QD_interior_ + QD_membrane_) (ratio: QD_interior_/QD_total_) using image processing tools on individual cells at different time points (See *Methods*) (Figure 5E). Only cells with similar IR-B-SYFP expression levels (estimated by the VFP intensity signal) were considered since high expression levels of IR affect internalization rates (data not shown). The data indicate that IGF-II internalized faster than insulin through IR-B. After short incubation at 37°C the fraction of internalized IGF-II-biot-QD655 (10 min: 0.60 ± 0.03; 20 min: 0.72 ± 0.03; *n* = 20 cells) was significantly higher than that of BAC-Ins-QD655 (10 min: 0.38 ± 0.03 and 20 min: 0.41 ± 0.03; *n* = 10–20 cells) in cells over-expressing IR-B (*p* < 0.00001). Differences between both ligands were also significant at the onset of the experiment. While insulin internalization at room temperature is negligible (0.06 ± 0.01), IGF-II is significantly endocytosed (0.17 ± 0.03) (*p* = 0.002; *n* = 28–29 cells). Data were fitted to a sigmoideal curve (logistic) and the first derivative in each time point was calculated finding the maximal k (k_max_). This analysis showed a higher k_max_ for IGF-II ((0.034 ± 0.002) min^-1^) than for insulin ((0.021 ± 0.001) min^-1^). At longer incubation times (150 min) both ligands are equally and almost completely internalized (IGF-II: 0.96 ± 0.01 and Insulin: 0.95 ± 0.01; *n* = 19–23 cells) (see Table 1).

**Table 1:** Internalization of IGF-II and insulin through IR-B by microscopy

**Time at 37°C**	**0 min**		**10 min**		**20 min**		**150 min**	
**Ligand**	**Insulin**	**IGF-II**	**Insulin**	**IGF-II**	**Insulin**	**IGF-II**	**Insulin**	**IGF-II**
**Average**	0.06	0.17	0.38	0.60	0.41	0.72	0.95	0.96
**s.e.m**	0.01	0.03	0.03	0.03	0.03	0.03	0.01	0.01
***n***	28	29	10	20	20	20	23	19
***p *(insulin versus IGF-II)**	2E-03		9E-06		7E-09		9E-01	

Quantification of the degree of internalization of IGF-II and insulin in HeLa cells expressing IR-B-SYFP labeled *in vivo *with 50 nM BAC-Ins and 1 nM QD655 at room temperature.

### IGF-II and insulin rate of internalization by flow cytometry

The results obtained by microscopy were confirmed measuring IGF-II internalization by flow cytometry detecting QD655 and SYFP signal. First, the specificity of QD655 binding to biotinylated ligands was evaluated in cells expressing IR-B or IR-B-SYFP and treated with QD655 with or without previous incubation with BAC-Ins, showing that the number of events with high QD655 signal was significantly higher when BAC-Ins was added (Additional file 6: Figure S5). On the other hand, the population of events showing high QD655 intensity shifted to the right if the cells expressed IR-B fused to a fluorescent protein confirming that only transfected cells are binding BAC-Ins-QD655 (Additional file 6: Figure S5). We defined four regions according to fluorescence intensity and quantified the events inside region II (high SYFP and QD655 signals) in cells expressing IR-B-SYFP treated with QD655 with or without BAC-Ins. We normalized these data to control cells without transfection (fold with respect to the control: 1.1 ± 0.1 for cells treated only with QD655 and 5.4 ± 0.3 for cells incubated with BAC-Ins and QD655; *n* = 3 independent experiments, *p* < 0.0005) confirming specific labeling (Additional file 6: Figure S5F).

**Figure 6:**
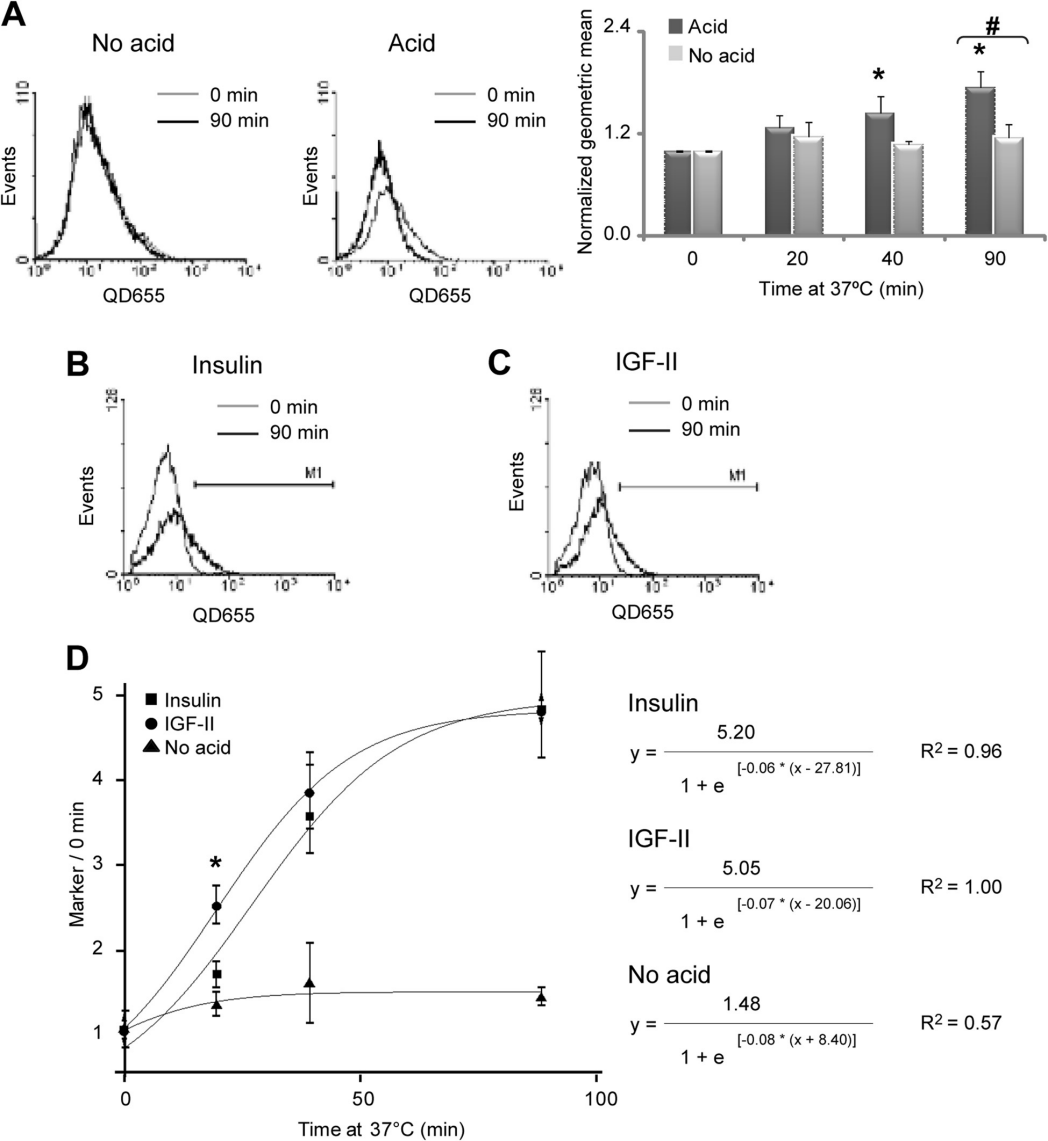
**Internalization of IGF-II and insulin through IR-B by flow cytometry. A**. HeLa cells expressing IR-B-SYFP labeled *in vivo *with 50 nM BAC-Ins and 1 nM QD655 at room temperature were incubated at 37°C for different times. Cells were treated or not with acid for 2 min, before collection with 0.5 mM EDTA in PBS and analyzed by flow cytometry detecting SYFP and QD655 signals. QD655 histograms were performed with the population of events which were SYFP positive (transfected cells). Overlays of histograms from cells incubated at 37°C for 90 min and non-incubated cells for each ligand treatment. In each histogram the geometric mean was calculated and was normalized with respect to the time defined as zero. Black asterisks show significant differences with the time defined as zero (*p* ≤ 0.05; *n* = 3 independent experiments). Numeral symbol indicates significant differences between acid and non acid treatments (*p* = 0.01; *n* = 3 independent experiments). **B-C**. Similar experiments with BAC-Ins (**B**) or IGF-II-biot (**C**). **D**. In each histogram a marker *M1* was determined including approximately 4% of the events at the time defined as zero. The percentage of events inside region *M1 *was measured and normalized with respect to the time defined as zero (Marker/0 min). The results are shown as the mean ± s.e.m (*n* = 5 independent experiments). Asterisk indicates significant differences between ligands (*p* = 0.01).

After confirming that flow cytometry allows us to detect specific binding of biotinylated ligands to IR-B in living and individual cells, we tested whether acid treatment could remove receptor-bound ligands at the plasma membrane. Cells expressing IR-B-SYFP were labeled with IGF-II-biot and QD655 and incubated with acid for 2 min. Living cells were analyzed by flow cytometry quantifying SYFP and QD655 signals. We estimated the ratio between the number of events showing high QD655 and SYFP signals (quadrant II) over the total events with high SYFP signal (quadrants II + IV; i.e., transfected cells) before and after acid treatment. The analysis showed that acid treatment removes the IGF-II-biot-QD655 bound to IR at the plasma membrane (Additional file 6: Figure S5G). Similar experiments were performed by incubating labeled cells for 20, 40 and 90 minutes at 37°C prior to acid treatment. The data were first analyzed by selecting the events with high SYFP signal (transfected cells) and generating QD655 histograms. The histograms showed that longer incubation at 37°C increased the QD655 geometric mean values, validating the cellular means as measures of internalized IGF-II-biot-QD655 (Figure 6A). While the geometric mean did not change in acid untreated cells, it did increase over the time in acid treated cells. Analysis of the histograms for IGF-II and insulin internalization over the time revealed a shift of the geometric mean of the QD655 signal (Figure 6B-C). We quantified the high QD655 signal events using a marker *M1* on each histogram including approximately 4% of the events at the time defined as zero (0 min). For each incubation point we determined the percentage of events inside the region *M1* and normalized it to the value obtained for the time defined as zero (Marker/0 min). According to the *M1* marker, a greater fraction of IR expressing cells internalized IGF-II-biot-QD655 than BAC-Ins-QD655. These differences were statistical significant (*p* = 0.01) after short incubation times (20 min), confirming the faster internalization dynamics induced by IGF-II than by insulin (Figure 6D; insulin: 1.7 ± 0.2 and IGF-II: 2.6 ± 0.2; *n* = 4 independent experiments). We fitted the data to a sigmoideal curve (logistic) and calculated the first derivatives. The analysis showed that the k_max_ for IGF-II was higher ((0.08 ± 0.02) min^-1^) than the k_max_ for insulin ((0.06 ± 0.01) min^-1^). In contrast, data from samples treated without acid did not show an increase over the time or a logistic fitting (R^2^ = 0.57).

## Discussion

We showed that IGF-II is a potent IR-B ligand inducing cell proliferation and AP-1 mediated gene transcription. Luciferase reporter assays confirmed that IGF-II could signal specifically through transfected IR since over-expression of IR-B turns HeLa cells responsive to rhIns and IGF-II, without affecting the induction promoted by EGF. Furthermore, while co-stimulation with EGF and rhIns triggered an additive AP-1 induction, rhIns + IGF-II did not exhibit a combined response. These results indicate that insulin and IGF-II induce AP-1 through the same IR/IGF-IR axis when IR is over-expressed, in contrast to EGF that signals independently.

The combination of IR-B fused to VFPs with biotinylated ligands as well as streptavidin conjugated QDs allow to visualize and quantitatively study IGF-II binding to the IR-B and its ensuing endocytosis. This approach permits to study endocytosis by tracking the ligand-receptor complex discriminating the internalized receptors from the cytoplasmatic pool. To label the ligand-receptor complexes exclusively at the cell surface we used a combination of biotinylated ligands and streptavidin conjugated QDs. Although this could also be reached using fluorescent streptavidin instead of QDs or fluorescently modified ligands, QDs provide powerful advantages for imaging: (a) high and uniform brilliance, allowing robust quantification of binding and transport processes, (b) photostability which is crucial for long term experiments, (c) broad excitation spectrum, allowing the co-imaging with VFPs without bleed-through risk, (d) narrow emission band in the red part of the spectra well separated from the fluorescence of the VFPs and (e) proper biochemical stability and specificity and low background [40]. Specificity of QD internalization was corroborated since in the absence of biotinylated ligands no QD signal was detected. We showed that internalization process using biotinylarted ligands + QDs occurs similarly than when using fluorescent insulin or biotinylated ligands + fluorescent streptavidin. The internalization of the complexes through endocytosis was also confirmed by co-localization with endocytosis markers during the process.

Additionally, the effect of biotinylation over ligand activity was controlled, biotinylated-IGF-II triggered IR-B signaling activation to a similar degree than human IGF-II measured as IR auto-phosphorylation and ERK 1/2 activation.

The endocytosis of IGF-II by IR-B exhibited to proceed with a faster kinetics than that of insulin measured both by confocal microscopy and flow cytometry. These techniques showed to be complementary since microscopy provides the advantage of visualizing the process in detail in individual cells and flow cytometry allows the analysis of a large number of events. By microscopy we measured internalization as the proportion of ligand-QD present inside individual cells expressing IR-B after different incubation times at 37°C. Complimentary, by flow cytometry we counted the number of cells exhibiting internalized ligand-QD655 after stimulation. We calculated the rate of internalization at the onset as well as after 10 min of incubation at 37°C leading us to similar results obtained by others using radioactive experiments [41-43]. Some of these reports are discordant depending on the cells used or the method of analysis applied. While for insulin internalization Draznin and colleagues [41] reported a k = 0.049 min^-1^ and a half time of 14.5 min, Knuston [44] discussed these results informing a k = 0.0023 and a half time of 4.9 hours. In our experiments, applying a completely different method, we obtain results closer to those reported by Draznin and colleagues [41]. In addition, our data confirm that, in IR-B over-expressing HeLa cells, IGF-II is internalized faster (half time = 20.3 min) than insulin (half time = 33.6 min). Recently, Morrione *et al.*[45] showed that insulin and IGF-II regulate endocytic sorting and stability of the IR-A. They showed that IGF-II induces lower IR-A activation than insulin. However, this differential endocytic sorting would protect IR-A from down-regulation, explaining a more sustained mitogenicity. This study shows, in an elegant manner, how one same receptor could be differentially regulated, triggering different signaling responses, after binding two different ligands. Although there are important discrepancies in the field, the overwhelming consensus is that IGF-II has low affinity for IR-B and high affinity for IR-A compared with that of insulin. We focused in the study of IR-B since this receptor is considered to be the one mostly responsible for the metabolic effect of insulin and we addressed its dynamics upon binding insulin (a metabolic regulator) and IGF-II (a mitogenic growth factor). Our interest was to address this open question *in vivo* and with new technologies that had not been applied yet to the insulin/IGF field.

## Conclusions

The evidence presented here is consistent with the model of signal transduction divergence modulated by the cellular localization of receptor-ligand complexes [46-55]. Mitogenic ligand-receptor complexes are internalized more rapidly, thereby triggering signaling from endosomes; whereas ligand-receptor complexes retained at the plasma membrane lead to a more metabolic response. This view is in accordance with a variety of reports using different experimental approaches. For example, insulin analogs exhibiting different receptor dissociation rates indicate that faster dissociating analogs lead to a more sustained activation of the receptor and a more persistent activation of Shc [53,56]. On the other hand, longer signal duration at the level of the receptor leads to a mitogenic response of insulin [56-59] suggesting that mitogenic signaling is mediated by a more sustained activation of the IR. Additionally, non-internalizing IR mutants showed an enhancement of some biological responses to insulin and attenuation of others [52] and pharmacological inhibition of endocytosis demonstrate that the metabolic actions of insulin are largely independent of insulin receptor endocytosis and are initiated by activation of the plasma membrane-localized insulin receptor [40]. We improved the duality of the studied model using one receptor and two ligands: a mitogenic one (IGF-II) and a metabolic one (insulin).

## Methods

### Materials

rhIns was kindly provided by Laboratorios Beta (Buenos Aires, Argentina). IGF-II-biot was from IBT system (Reutlingen, Germany) and human recombinant IGF-II was from Gro Pep (Adelaide, Australia). BAC-Ins was from Sigma (Munich, Germany). Polyclonal rabbit anti-ERK2, polyclonal rabbit anti-CD63 (Lamp 1) and polyclonal rabbit anti-IR-α-subunit (N20 and H78) were from Santa Cruz Biotechnology (Paso Robles, CA). EEA1 marker (rabbit) was from Abcam (Cambridge, MA). Monoclonal mouse anti-phosphorylated-tyrosine (PY20) was from BD Transduction Laboratories (Franklin Lakes, NJ). Monoclonal rabbit anti-phosphorylated-IR-β subunit (Tyrosine 1361), monoclonal mouse anti-IR-β subunit and polyclonal rabbit anti-phosphorylated (p42/44) MAPK were from Cell Signaling Technology (Beverly, MA). The monoclonal rabbit anti-phosphorylated-IR-β subunit (Tyrosine 1361) from Cell Signaling (cat # 3023) only detects transfected levels of IR β subunit and slightly cross-reacts with activated IGF-I receptor. The streptavidin conjugated QD655 and secondary antibodies conjugated with Alexa fluor 555 were from Molecular Probes, Invitrogen (Eugene, OR). Streptavidin-atto 550 was from ATTO-TEC Gmbh (Siegen, Germany). Lipofectamine Reagent 2000, DNase I and Trizol were from Invitrogen (Carlsbad, CA). High Capacity cDNA Reverse Transcription kit was from Applied Biosystem (Foster City, CA). Cell Titer 96 Aqueous Non-Radioactive Cell Proliferation Assay (MTS) and Gotaq DNA polymerase were from Promega (Madison, WI). The plasmid containing the luciferase reporter gene downstream of seven binding sites for AP-1 (pAP-1-Luc) was provided by Dr. Omar Coso (IFIBYNE, Argentina).

### Plasmids generation

*pcDNA3-IR-B*: cDNA of IR-B was provided by Dr. Axel Ullrich (Max Planck Institute for Biophysical Chemistry, Germany) and amplified by PCR using primers containing HindIII restriction site (IR-Forward: 5′-aagcttatggccaccgggggccgg-3′) and NheI and XbaI sites (IR-Reverse: 5′-tctagagctagcgaaggattggaccgaggcaaggtc-3′). The product was digested with HindIII and XbaI enzymes and cloned into the pcDNA3 vector. *pcDNA3-IR-B-GFP, pcDNA3-IR-B-SCFP and pcDNA3-IR-B-SYFP:* peGFP-C3 (Clontech), pSCFP-3AC or pSYFP-2F (provided by T. W. Gadella Jr., University of Amsterdam, The Netherlands) were digested with NheI and ApaI and cloned into pcDNA3-IR-B.

### Cell culture and transfections

Cell culture reagents (Dulbecco’s modified Eagle’s medium (DMEM) Glutamax, Optimem, fetal bovine serum (FBS), trypsin and antibiotics) were from GIBCO (Grand Island, NE). HeLa cells were maintained in DMEM supplemented with penicillin, streptomycin and 10% FBS at 37°C in 5% CO_2_. Human cervical carcinoma HeLa cells (ATCC-CCL-2) were plated at 1×10^5^ cells/well in 24 well plates and onto 12 mm glass coverslips (for microscopy experiments) or at 2.5×10^5^ cells/well in 12 wells plates (for Western blot experiments) one day before transfection in DMEM supplemented with 10% FBS without antibiotics. Cells were transfected with Lipofectamine Reagent 2000 for 5 hours using 0.4 μg DNA and 1 μl Lipofectamine per well for 24 well plates and 0.8 μg DNA and 2 μl Lipofectamine per well for 12 well plates. After transfection cells were cultured for further expression in complete medium.

### Western blots

Following stimulation with rhIns, BAC-Ins, IGF-II or IGF-II-biot cells were lysed in buffer containing 100 mM Tris–HCl pH 6.8, 4% SDS, 0.2% bromophenol blue, 20% glycerol, 200 mM β-mercaptoethanol, vortexed for 20 sec and heated 5 min at 100°C. After 10% SDS-PAGE and transfer, membranes were blocked in 5% w/v non-fat dried milk in 0.1% Tween-TBS buffer (TTBS) for 1 hour, washed and incubated overnight at 4°C with primary antibodies diluted in 5% bovine serum albumin (BSA)/TTBS (anti-IR-β subunit: 1/500, anti-ERK2: 0.2 μg/ml, anti-phosphorylated-p44/42 MAPK: 1/500) or in 2% BSA/TTBS (anti-phosphorylated-Tyrosine: 2 μg/ml). Membranes were incubated with secondary antibodies for 1 hour and washed with TTBS. The blots were developed by chemiluminescence with a Bio-Imaging Analyzer Bas-1800II and Image Gauge 3.12, FUJIFILM.

### Luciferase reporter assay

Cells seeded onto 24 well plates (1 × 10^5^ cells/well) the day before were transfected using 0.3 μg pcDNA3-IR or pcDNA3 (empty vector, EV) and 0.05 μg pAP1-Luc. After 24 hours, cells were starved one day, and then stimulated for 16 hours with 1.6 nM EGF, 100 nM rhIns, 100 nM IGF-II, 1.6 nM EGF + 100nM rhIns or 100 nM IGF-II + 100 nM rhIns. Luciferase activity was determined using Luciferase Reactive and Reporter Lysis Buffer from Promega (Madison, WI) and normalization to the control (non-stimulated cells) was performed (fold induction). The results were expressed as the mean of at least three independent experiments ± s.e.m. The *p* values were estimated using Student’s *T* test (2 tails).

### Cell proliferation experiments

Cells were transfected with pcDNA3-IR or the EV, 24 hours after transfection they were treated with 1% FBS overnight before stimulation with 0.1 nM, 1 nM or 10 nM rhIns or IGF-II for 2 days. Cell proliferation was measured using a non-radioactive assay (MTS) according to manufacturer procedures. The results were normalized to those obtained for the cells incubated in 1% FBS over all the experiments. The results were expressed as the mean of at least three independent experiments ± s.e.m. The *p* values were estimated using Student’s *T* test (2 tails).

### Labeling *in vivo* with QDs-IGF-II-biot or BAC-Ins and internalization

Before the experiment cells expressing IR-B, IR-B-SYFP or IR-B-GFP were starved overnight, washed with Tyrode’s buffer (135 mM NaCl, 10 mM KCl, 10 mM MgCl_2_, 1mM CaCl_2_, 10 mM HEPES pH 7.2, 0.1% BSA) at room temperature or 15°C (as indicated in figure legends) and incubated with 50 nM BAC-Ins or IGF-II-biot for 15 min, washed with Tyrode’s and incubated with 0.5, 1.0, 2.0 or 4.0 nM QD655 for 10 min in darkness, washed and either fixed in 3.7% paraformaldehyde (PFA) on ice for 20 min or incubated at 37°C in DMEM for different periods of time before fixation. When acid treatment was applied, cells were incubated for 5 min (microscopy) or 2 min (flow cytometry) at room temperature with, 0.5 M NaCl, 0.1 M Na-glycine pH 3.0 and then fixed in 3.7% PFA for microscopy experiments or directly analyzed by flow cytometry.

### Immunofluorescence

After overnight starvation, transfected cells were stimulated with 100 nM rhIns, IGF-II or IGF-II-biot for 5 min, washed with cold PBS and immediately fixed in cold methanol for 30 min at -20°C, blocked with PBS/0.3% Triton X-100/1% BSA for 1 hour at 37°C and incubated with anti-phosphorylated-IR-β subunit (0.3 μg/ml) overnight at 4°C. The following day the samples were incubated with a secondary antibody conjugated with Alexa fluor 555 for 1 hour at 37°C and washed. IR activation was monitored by confocal microscopy. To study the expression of IR-B-GFP we followed the same strategy but we did not starve the cells before the assay. We used anti-IR-α-subunit (N20 and H78) (0.2 μg/ml), the following day we incubated the cells with a secondary antibody conjugated with Cy3. For endocytosis immunofluorescence, HeLa cells expressing IR-B were labeled with BAC-Ins and QD655, induced internalization and fixed in 3.7% PFA. Immunofluorescences were performed with 4 μg/ml anti-CD63 or 1.5 μg/ml EEA1.

### Microscopy

Confocal laser scanning microscopy was performed with: *(i)* an Olympus Fluoview FV 1000 microscope with a UPLSAPO 60× 1.2 NA water immersion objective. Excitation and emission filters were as follows: excitation SCFP, 405 nm; emission SCFP, band pass (BP): 430–470 nm; excitation GFP, 488 nm; emission GFP, BP: 505–605 nm; excitation SYFP, 515 nm; emission SYFP, BP: 535–565 nm; excitation DAPI, 405 nm; emission DAPI, BP: 430–470 nm; excitation QD655, 405 nm; emission QD655, BP: 655–755 nm; excitation Alexa fluor 555, 543 nm; emission Alexa fluor 555, BP: 560–620 nm; *(ii)* a Zeiss LSM 510 Meta microscope with a Plan-Apochromat 63× 1.4 NA oil immersion objective. Excitation and emission filters were as follows: excitation FITC, 488 nm; emission FITC, 510–563 nm.

We always used the sequential mode for image acquisition.

In the experiments where imaging was performed with a Zeiss LSM 510 Meta microscope we used a C-Apochromat 6× 1.2 NA water immersion objective. Excitation and emission filters were as follows: excitation GFP, 488 nm; emission GFP, BP: 500/20 nm; excitation QD655, 488 nm and 458 nm; emission QD655, LP: 650 nm.

Wide field microscopy was performed with a Zeiss Axiovert S100 with a 63× 1.25 NA oil immersion objective (Zeiss), a mercury arc lamp excitation and filters suitable for GFP, Cy3 and DAPI signals. Camera: Hamamatsu Orca CCD C4742-95.

### Image processing

Confocal microscope images were processed with Matlab (TU Delft, The Netherlands) and Image J (National Institutes of Health). The background of each channel (mean of empty region) was subtracted and in some cases a median filter was applied (radius: 1 pixel) only for presentation. No filter was applied in quantitative analyses. The *z*-stacks (64 frames, 0.16 μm step size) were processed by deconvolution using Scientific Volume Imaging Huygens Professional Version 3.6 software, applying a Quick Maximum Likelihood Estimation (QMLE) algorithm.

### Internalization analysis

For quantitative internalization experiments, we defined time “0 min” as the end of labeling-washes, and t = 10, 20 and 150 minutes of incubation at 37°C.

#### Segmentation (membrane and interior)

Channel backgrounds (median) were subtracted. Segmentation was performed for each cell using the SYFP signal. After *cell* segmentation the *pre-membrane* was defined as the difference image of the cell and a binary erosion (iterations: 5–20; alternating connectivity); we evaluated the results visually. The *pre-interior* was defined as the difference between the *cell* and the *pre-membrane*. A *QD*_*mask*_ marked red pixels. With this mask a *membrane* was defined as the product of the *QD*_*mask*_ and *pre-membrane*, and *interior* as the product of the *QD*_*mask*_ and *pre-interior* obtained previously.

#### Estimation of the relative amount of internalization

Values in *membrane* and *interior* were summed for red and green channel, and also sizes were measured. To compute the relative amount of internalized red fluorescence we estimated QD_*total*_ as the sum of QD_*membrane*_ and QD_*interior*_ and we calculated for each cell the ratio QD_*interior*_/QD_*total*_. This “internal calibration” approach was chosen to remove the influence of the amplifier gain and the zoom factor for each image acquisition condition. The expression levels were estimated as the mean of the SYFP signal (sum of SYFP/cell size) and for the statistical analysis only cells with similar level of IR-SYFP expression were considered.

### Colocalization analysis

Manders coefficients and PDM graphs were performed with the Image J plugin Intensity correlation analysis. The PDM graphs show the contribution of each pixel to the colocalization coefficient.

### Flow cytometry

Flow cytometry was performed with a Becton Dickinson FACSAriaII. The filters, lasers and dichroic mirrors (DM) were as follow: *(i)* SYFP: excitation 488 nm, DM LP: 502 nm, emission BP: 530/30 nm; *(ii)* QD655: excitation 488 nm, DM LP: 655 nm, emission BP: 660/20 nm. Data analysis was performed with WinMDI Version 2.9 software. Positive events for SYFP signal were selected taking into account basal auto-fluorescence of control cells (non-transfected cells). QD655 histograms were performed with the population of events SYFP positive (transfected cells) and overlays of histograms from cells incubated at 37°C for 20 or 90 min and non-incubated cells were displayed. A geometric mean was calculated for each histogram using the WinMDI. In each histogram a marker *M1* was determined including approximately 4% of the events at the initial moment (0 min). For each internalization period the percentage of events inside region M1 was measured and the normalization was done with respect to the initial step (0 min) (Marker/0 min).

### Graphical fitting

The data for internalization experiments (microscopy and flow cytometry) were fitted by Origin 8.6 using sigmoideal logistic curve.

### Statistical analysis

The results were expressed as the mean ± s.e.m. *p* values were estimated using two-tailed Student’s t-tests.

For supplementary methods see Additional file 7.

## Abbreviations

BAC-Ins: Biotin amido caproyl insulin; BP: Band-pass; BSA: Bovine serum albumin; DM: Dichroic mirror; EEA1: Early endosomes marker; EGF: Epidermal growth factor; EV: Empty vector; FBS: Fetal bovine serum; GFP: Enhanced green fluorescent protein; IGF: Insulin like growth factor; IGF-II-biot: Biotinylated IGF-II; IGF-IR: Insulin like growth factor I receptor; IR: Insulin receptor; IR-A: Insulin receptor isoform A; IR-B: Insulin receptor isoform B; M: Manders coefficient; PDM: Differences from the mean; PFA: Paraformaldehyde; QD: Quantum dot; rhIns: Recombinant human insulin; QMLE: Quick maximum likelihood estimation algorithm; SA: Streptavidin; SCFP: Super cyan fluorescent protein; s.e.m: Standard error of the mean; SYFP: Super yellow fluorescent protein; VFP: Visible fluorescent protein.

## Competing interests

The authors declare that they have no competing interests.

## Authors’ contributions

JG conceived, designed and performed the experiments and deconvolution analysis, analyzed the data and wrote the paper. LSB designed and performed experiments. FFG carried out deconvolution analysis. APS sequenced and analyzed all DNA plasmids. LG revised the manuscript critically. EJE conceived and designed the experiments, analyzed the data and wrote the paper. FCL conceived and designed the experiments, analyzed the data and wrote the paper. All authors read and approved the final manuscript with the exception of the deceased, Elizabeth Jares Erijman.

## Supplementary Material

Additional file 1: Figure S1 IR and IGF-IR expression in HeLa cells. mRNA from HeLa cells was reverse-transcribed and assayed by PCR (RT-PCR). A. Splicing of exon 11 (36 nt) was assayed using primers that annealed in the flanking constitutive regions (i.e. exon 10 and exon 12). PCR products were analyzed by 6% PAGE and percentage of exon inclusion was quantified by densitometry. B. mRNA levels of IR and IGF-IR were assayed by RT-PCR with different amounts of cDNA. Quantification was performed by densitometry in the region where the response was linear. The results are expressed as the mean ± s.e.m (*n* = 2 independent experiments). C. PCR showing IR-B over-expressed in HeLa cells. Click here for file

Additional file 2: Figure S2 Estimation of transfection efficiency. HeLa cells were transfected with pcDNA3-IR-B-GFP (A) or pcDNA3-IR-B (B) and labeled with 50 nM BAC-Ins for 15 min and 1nM QD655 for 10 min. After labeling cells were imaged by confocal microscopy (Zeiss LSM 510 Meta). Scale bars: 10 μm. **C. **Total number of cells was estimated from DIC images. Transfected cells were estimated from GFP images for IR-B-GFP and from QD655 images for IR-B. Results are expressed as the mean ± s.e.m (*n* = 219 cells for IR-VFP and *n* = 308 cells for IR from at least 3 images from 3 independent experiments). Click here for file

Additional file 3: Figure S3 Expression of IR-B-GFP by immunofluorescence. HeLa cells expressing IR-B-GFP were fixed in methanol and then incubated with primary antibodies against two different regions of the N-terminus of the α-subunit of IR (N20 and H78). Secondary antibody was conjugated with Cy3. Imaging was carried out by epifluorescence microscopy (Zeiss Axiovert S100). Scale bars: 5 μm. Click here for file

Additional file 4: Figure S4 Internalization of FITC-Insulin and BAC-Ins-SA-atto 550. A. HeLa cells over-expressing IR-B-GFP were labeled with 50 nM BAC-Ins for 15 min and then with 1 nM SA-atto 550 for 10 min at RT. Cells were directly fixed in PFA (upper panel) or incubated at 37°C for 60 min before fixation (middle panel). Lower panel shows the control experiment where the cells were treated similarly but without incubation with BAC-Ins. Imaging was performed by confocal microscopy (Olympus Fluoview FV1000). Scale bars: 10 μm. B. HeLa cells transfected with pcDNA3-IR-B were labeled with 50 nM FITC-insulin for 15 min and then directly fixed in methanol (upper panel) or incubated at 37°C for 75 min before fixation (middle panel). Lower panel shows similar experiment but in non transfected HeLa cells. Imaging was performed by confocal microscopy (Zeiss LSM 510 Meta). Scale bars: 10 μm. Click here for file

Additional file 5: Movie S1 Visualization of IGF-II-biot-QD655 bound to IR-B-GFP and endocytosed. HeLa cells expressing IR-B-GFP were labeled *in vivo *with 50 nM IGF-II-biot and 2 nM QD655, incubated at 37°C for 1 hour, treated for 5 min with 0.1 mM Na-glycine pH 3.0, NaCl 0.5 M and fixed in 3.7% PFA. Images were taken with a confocal microscope by acquisition of 64 z-slices with 16 μm step size. Deconvolution was performed with Huygens Scientific Volume Imaging Huygens Professional Version 3.6, applying the Quick maximum likelihood estimation algorithm. The movie shows tridimensional reconstruction using the surface renderer process observed from different angles. Click here for file

Additional file 6: Figure S5 Specificity of the binding of BAC-Ins and IGF-II-biot to the IR-B by flow cytometry. HeLa cells over-expressing IR-B (A-C) or IR-B-SYFP (D and E) were labeled *in vivo *with 50 nM BAC-Ins and 1 nM QD655 (C and E) or only with 1 nM QD655 (B and D). Cells were collected with 0.5 mM EDTA in PBS and were analyzed by flow cytometry detecting SYFP and QD655 signals. The images inside the graphs correspond to similar experiments but analyzed by confocal microscopy (QD655 are shown in red and SYFP in green). F. Quantification of the proportion of events in the region II (*see panel A*) with high signal of SYFP (transfected cells) and high signal of QD655 (insulin or IGFII binding). We normalized this value to the proportion obtained for the cells only incubated with QD655 without biotinylated ligand. G. HeLa cells expressing IR-B-SYFP were labeled *in vivo *with 50 nM IGF-II-biot and 1 nM QD655 at room temperature and were treated (or not) with acid (0.1 M Na-glycine pH 3, 0.5 M NaCl) for 2 min. After washing with PBS, cells were collected with 0.5 mM EDTA in PBS and analyzed by flow cytometry detecting SYFP and QD655 signals. The bar graph shows the quantification of the ratio between the events inside region II and the events inside region (II + IV). Asterisks indicate significant differences (*p* ≤ 0.001; *n* = 3 independent experiments). Click here for file

Additional file 7 Supplementary methods. Click here for file

## References

[B1] Chan SJ Steiner DF Insulin through the ages: phylogeny of a growth promoting and metabolic regulatory hormone Am Zool 2000 40 213 222 10.1668/0003-1569(2000)040[0213:ITTAPO]2.0.CO;2

[B2] Kimura KD Tissenbaum HA Liu Y Ruvkun G daf-2, an insulin receptor-like gene that regulates longevity and diapause in Caenorhabditis elegans Science 1997 277 942 946 10.1126/science.277.5328.942 9252323

[B3] Efstratiadis A Genetics of mouse growth Int J Dev. Biol 1998 42 955 976 9853827

[B4] Tissenbaum HA Ruvkun G An insulin-like signaling pathway affects both longevity and reproduction in Caenorhabditis elegans Genetics 1998 148 703 717 9504918 10.1093/genetics/148.2.703 PMC1459840

[B5] Brogiolo W Stocker H Ikeya T Rintelen F Fernandez R An evolutionarily conserved function of the Drosophila insulin receptor and insulin-like peptides in growth control Curr Biol 2001 11 213 221 10.1016/S0960-9822(01)00068-9 11250149

[B6] Nakae J Kido Y Accili D Distinct and overlapping functions of insulin and IGF-I receptors Endocr Rev 2001 22 818 835 10.1210/er.22.6.818 11739335

[B7] Saltiel AR Kahn CR Insulin signalling and the regulation of glucose and lipid metabolism Nature 2001 414 799 806 10.1038/414799a 11742412

[B8] Holzenberger M Dupont J Ducos B Leneuve P Geloen A IGF-1 receptor regulates lifespan and resistance to oxidative stress in mice Nature 2003 421 182 187 10.1038/nature01298 12483226

[B9] Nef S Verma-Kurvari S Merenmies J Vassalli JD Efstratiadis A Testis determination requires insulin receptor family function in mice Nature 2003 426 291 295 10.1038/nature02059 14628051

[B10] Fernández R Tabarini D Azpiazu N Frasch M Schlessinger J The Drosophila insulin receptor homolog: a gene essential for embryonic development encodes two receptor isoforms with different signaling potential EMBO J 1995 14 3373 3384 7628438 10.1002/j.1460-2075.1995.tb07343.x PMC394404

[B11] Pashmforoush M Chan SJ Steiner DF Structure and expression of the insulin-like peptide receptor from amphioxus Mol Endocrinol 1996 10 857 866 10.1210/me.10.7.857 8813726

[B12] Ruvkun G Hobert O The taxonomy of developmental control in Caenorhabditis elegans Science 1998 282 2033 2041 9851920 10.1126/science.282.5396.2033

[B13] Ebina Y Ellis L Jarnagin K Edery M Graf L The human insulin receptor cDNA: the structural basis for hormone-activated transmembrane signalling Cell 1985 40 747 758 10.1016/0092-8674(85)90334-4 2859121

[B14] Ullrich A Bell JR Chen EY Herrera R Petruzelli LM Human insulin receptor and its relationship to the tyrosine kinase family of oncogenes Nature 1985 313 756 761 10.1038/313756a0 2983222

[B15] Ullrich A Gray A Tam AW Yang-Feng T Tsubokawa M Insulin-like growth factor I receptor primary structure: comparison with insulin receptor suggests structural determinants that define functional specificity EMBO J 1986 5 2503 2512 2877871 10.1002/j.1460-2075.1986.tb04528.x PMC1167146

[B16] Shier P Watt VM Primary structure of a putative receptor for a ligand of the insulin family J Biol Chem 1989 264 14605 14608 2768234

[B17] De Pirro R Forte F Bertoli A Greco AV Lauro R Changes in insulin receptors during oral contraception J Clin Endocrinol Metab 1991 52 29 33 7451643 10.1210/jcem-52-1-29

[B18] Mosthaf L Vogt B Haring HU Ullrich A Altered expression of insulin receptor types A and B in the skeletal muscle of non-insulin-dependent diabetes mellitus patients Proc Natl Acad Sci USA 1991 88 4728 4730 10.1073/pnas.88.11.4728 1711209 PMC51739

[B19] Kellerer M Sesti G Seffer E Obermaier-Kusser B Pongratz DE Altered pattern of insulin receptor isotypes in skeletal muscle membranes of type 2 (noninsulin-dependent) diabetic subjects Diabetologia 1993 36 628 632 10.1007/BF00404072 8359580

[B20] Savkur RS Philips AV Cooper TA Aberrant regulation of insulin receptor alternative splicing is associated with insulin resistance in myotonic dystrophy Nat Genet 2001 29 40 47 10.1038/ng704 11528389

[B21] Yakar S Liu JL Stannard B Butler A Accili D Normal growth and development in the absence of hepatic insulin-like growth factor I Proc Natl Acad Sci USA 1999 96 7324 7329 10.1073/pnas.96.13.7324 10377413 PMC22084

[B22] Sutter NB Bustamante CD Chase K Gray MM A single IGF1 allele is a major determinant of small size in dogs Science 2007 316 112 115 10.1126/science.1137045 17412960 PMC2789551

[B23] Guevara-Aguirre J Balasubramanian P Guevara-Aguirre M Wei M Madia F Growth hormone receptor deficiency is associated with a major reduction in Pro-aging signaling, cancer, and diabetes in humans Sci Transl Med 2011 3 70ra13 10.1126/scitranslmed.3001845 PMC3357623 21325617

[B24] Whittaker J Groth AV Mynarcik DC Pluzek L Gadsboll VL Whittaker LJ Alanine scanning mutagenesis of a type 1 insulin-like growth factor receptor ligand binding site J Biol Chem 2001 276 43980 43986 10.1074/jbc.M102863200 11500492

[B25] De Meyts P Insulin and its receptor: structure, function and evolution Bioessays 2004 26 1351 1362 10.1002/bies.20151 15551269

[B26] Papa V Gliozzo B Clark GM McGuire WL Moore D Insulin-like growth factor-I receptors are overexpressed and predict a low risk in human breast cancer Cancer Res 1993 53 3736 3740 8339284

[B27] Pandini G Vigneri R Costantino A Frasca F Ippolito A Insulin and insulin-like growth factor-I (IGF-I) receptor overexpression in breast cancers leads to insulin/IGF-I hybrid receptor overexpression: evidence for a second mechanism of IGF-I signaling Clin Cancer Res 1999 5 1935 1944 10430101

[B28] Vella V Sciacca L Pandini G Mineo R Squatrito S The IGF system in thyroid cancer: new concepts Mol Pathol 2001 54 121 124 10.1136/mp.54.3.121 11376121 PMC1187048

[B29] Kasuga M Fujita-Yamaguchi Y Blithe DL White MF Kahn CR Characterization of the insulin receptor kinase purified from human placental membranes J Biol Chem 1983 258 10973 10980 6309826

[B30] Soos MA Siddle K **Immunological relationships between receptors for insulin and insulin-like growth factor I. Evidence for structural heterogeneity of insulin-like growth factor I receptors involving hybrids with IRs.** Biochem J 1989 263 553 563 2480779 10.1042/bj2630553 PMC1133463

[B31] Soos MA Whittaker J Lammers R Ullrich A Siddle K **Receptors for insulin and insulin-like growth factor-I can form hybrid dimers. Characterisation of hybrid receptors in Transfected cells.** Biochem J 1990 270 383 390 1698059 10.1042/bj2700383 PMC1131733

[B32] Soos MA Field CE Siddle K Purified hybrid insulin/insulin-like growth factor-I receptors bind insulin-like growth factor-I, but not insulin, with high affinity Biochem J 1993 290 419 426 8452530 10.1042/bj2900419 PMC1132290

[B33] Yamaguchi Y Flier JS Yokota A Benecke H Backer JM Moller DE Functional properties of two naturally occurring isoforms of the human insulin receptor in Chinese hamster ovary cells Endocrinology 1991 129 2058 2066 10.1210/endo-129-4-2058 1655392

[B34] Benyoucef S Surinya KH Hadaschik D Siddle K Characterization of insulin/IGF hybrid receptors: contributions of the insulin receptor L2 and Fn1 domains and the alternatively spliced exon 11 sequence to ligand binding and receptor activation Biochem J 2007 403 603 613 10.1042/BJ20061709 17291192 PMC1876384

[B35] Pandini G Frasca F Mineo R Sciacca L Vigneri R Belfiore A Insulin/insulin-like growth factor I hybrid receptors have different biological characteristics depending on the insulin receptor isoform involved J Biol Chem 2002 277 39684 39695 10.1074/jbc.M202766200 12138094

[B36] Denley A Bonython ER Booker GW Cosgrove LJ Forbes BE Structural determinants for high-affinity binding of insulin-like growth factor II to insulin receptor (IR)-A, the exon 11 minus isoform of the IR Mol Endocrinol 2004 18 2502 2512 10.1210/me.2004-0183 15205474

[B37] Sciacca L Costantino A Pandini G Mineo R Frasca F Insulin receptor activation by IGF-II in breast cancers: evidence for a new autocrine/paracrine mechanism Oncogene 1999 18 2471 2479 10.1038/sj.onc.1202600 10229198

[B38] Pandini G Medico E Conte E Sciacca L Vigneri R Belfiore A Differential gene expression induced by insulin and insulin-like growth factor-II through the insulin receptor isoform A J Biol Chem 2003 278 42178 42189 10.1074/jbc.M304980200 12881524

[B39] Pastore S Mascia F Mariotti F Dattilo C Mariani V Girolomoni G ERK1/2 regulates epidermal chemokine expression and skin inflammation J Immunol 2005 174 5047 5056 15814736 10.4049/jimmunol.174.8.5047

[B40] Lidke DS Nagy P Heintzmann R Arndt-Jovin DJ Post J Grecco H Quantum dot ligands reveal EGFR dynamics in living cells Nat Biotechnol 2004 22 198 203 10.1038/nbt929 14704683

[B41] Draznin B Trowbridge M Ferguson L Quantitative studies of the rate of insulin internalization in isolated rat hepatocytes Biochem J 1984 218 307 312 6370239 10.1042/bj2180307 PMC1153342

[B42] Hachiya HL Takayama S White MF King GL Regulation of insulin receptor internalization in vascular endothelial cells by insulin and phorbol ester J Biol Chem 1987 262 6417 6424 3106355

[B43] Formisano P Najjar SM Gross CN Philippe N Oriente F Kern-Buell CL Accili D Gorden P Receptor-mediated internalization of insulin. Potential role of pp120/HA4, a substrate of the insulin receptor kinase J Biol Chem 1995 270 24073 24077 10.1074/jbc.270.41.24073 7592607

[B44] Knutson VP Ligand-independent internalization and recycling of the insulin receptor. Effects of chronic treatment of 3T3-C2 fibroblasts with insulin and dexamethasone J Biol Chem 1992 267 931 937 1730683

[B45] Morcavallo A Genua M Palummo A Kletvikova E Jiracek J Brzozowski AM Iozzo RV Belfiore A Morrione A Insulin and Insulin-like Growth Factor II Differentially regulate endocytic sorting and stability of insulin receptor isoform A J Biol Chem 2012 287 11422 11436 10.1074/jbc.M111.252478 22318726 PMC3322853

[B46] Sorkin A Eriksson A Heldin CH Westermark B Claesson-Welsh L Pool of ligand bound platelet-derived growth factor beta-receptors remain activated and tyrosine phosphorylated after internalization J Cell Physiol 1993 156 373 382 10.1002/jcp.1041560221 7688373

[B47] Bergeron JJ Di Guglielmo GM Baass PC Authier F Posner BI Endosomes, receptor tyrosine kinase internalization and signal transduction Biosci Rep 1995 15 411 418 10.1007/BF01204345 9156572

[B48] Biener Y Feinstein R Mayak M Kaburagi Y Kadowaki T Zick Y **Annexin II is a novel player in insulin signal transduction. Possible association between annexin II phosphorylation and insulin receptor internalization.** J Biol Chem 1996 271 29489 29496 10.1074/jbc.271.46.29489 8910617

[B49] Grimes ML Zhou J Beattie EC Yuen EC Hall DE Endocytosis of activated TrkA: evidence that nerve growth factor induces formation of signaling endosomes J Neurosci 1996 16 7950 7964 8987823 10.1523/JNEUROSCI.16-24-07950.1996 PMC6579208

[B50] Ceresa BP Kao AW Santeler SR Pessin JE Inhibition of clathrin mediated endocytosis selectively attenuates specific insulin receptor signal transduction pathways Mol Cell Biol 1998 18 3862 3870 9632770 10.1128/mcb.18.7.3862 PMC108970

[B51] Parpal S Karlsson M Thorn H Straǻlfors P Cholesterol depletion disrupts caveolae and insulin receptor signaling for metabolic control via insulin receptor substrate- 1, but not for mitogen-activated protein kinase control J Biol Chem 2001 276 9670 9678 10.1074/jbc.M007454200 11121405

[B52] Hamer I Foti M Emkey R Cordier-Bussat M Philippe J An arginine to cysteine (252) mutation in insulin receptors from a patient with severe insulin resistance inhibits receptor internalization but preserves signalling events Diabetologia 2002 45 657 667 10.1007/s00125-002-0798-5 12107746

[B53] Jensen M Hansen B De Meyts P Schäffer L Ursø B Activation of the insulin receptor by insulin and a synthetic peptide leads to divergent metabolic and mitogenic signaling and responses J Biol Chem 2007 282 35179 35186 10.1074/jbc.M704599200 17925406

[B54] Uhles S Moede T Leibiger B Berggren PO Leibiger IB Selective gene activation by spatial segregation of insulin receptor B signaling FASEB J 2007 21 1609 1621 10.1096/fj.06-7589com 17264162

[B55] Jensen M De Meyts P Molecular mechanisms of differential intracellular signaling from the insulin receptor Vitam Horm 2009 80 51 75 19251034 10.1016/S0083-6729(08)00603-1

[B56] Hansen BF Danielsen GM Drejer K Sørensen AR Wiberg FC Sustained signalling from the insulin receptor after stimulation with insulin analogues exhibiting increased mitogenic potency Biochem J 1996 315 Pt 1 271 279 8670118 10.1042/bj3150271 PMC1217182

[B57] Ish-Shalom D Christoffersen CT Vorwerk P Sacerdoti-Sierra N Shymko RM Mitogenic properties of insulin and insulin analogues mediated by the insulin receptor Diabetologia 1997 2 Suppl 40 S25 S31 9248698 10.1007/s001250051393

[B58] De Meyts P Shymko RM Timing-dependent modulation of insulin mitogenic versus metabolic signalling Novartis Found Symp 2000 227 46 57 10752064 10.1002/0470870796.ch4

[B59] Kurtzhals P Schäffer L Sørensen A Kristensen C Jonassen I Correlations of receptor binding and metabolic and mitogenic potencies of insulin analogs designed for clinical use Diabetes 2000 49 999 1005 10.2337/diabetes.49.6.999 10866053

