# Supplementary figures and images for: Insulin and insulin like growth factor II endocytosis and signaling via insulin receptor B
Source: Cell Commun Signal. 2013 Mar 11;11:18. doi: 10.1186/1478-811X-11-18 (PMC3607927; doi:10.1186/1478-811X-11-18)

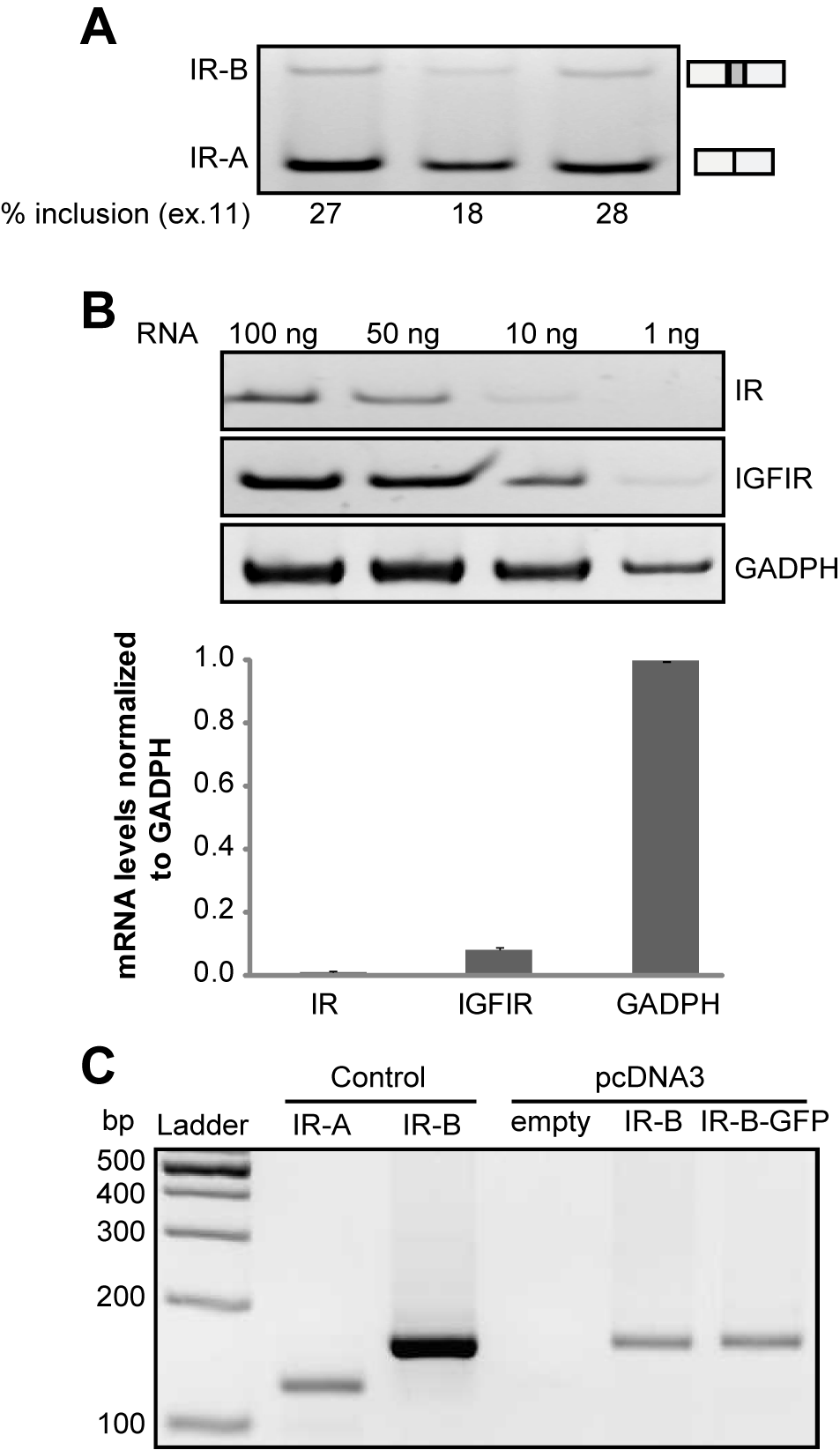

Supplement: Additional file 1: Figure S1 — IR and IGF-IR expression in HeLa cells. mRNA from HeLa cells was reverse-transcribed and assayed by PCR (RT-PCR). A. Splicing of exon 11 (36 nt) was assayed using primers that annealed in the flanking constitutive regions (i.e. exon 10 and exon 12). PCR products were analyzed by 6% PAGE and percentage of exon inclusion was quantified by densitometry. B. mRNA levels of IR and IGF-IR were assayed by RT-PCR with different amounts of cDNA. Quantification was performed by densitometry in the region where the response was linear. The results are expressed as the mean ± s.e.m (n = 2 independent experiments). C. PCR showing IR-B over-expressed in HeLa cells. [file 1478-811X-11-18-S1.tiff]

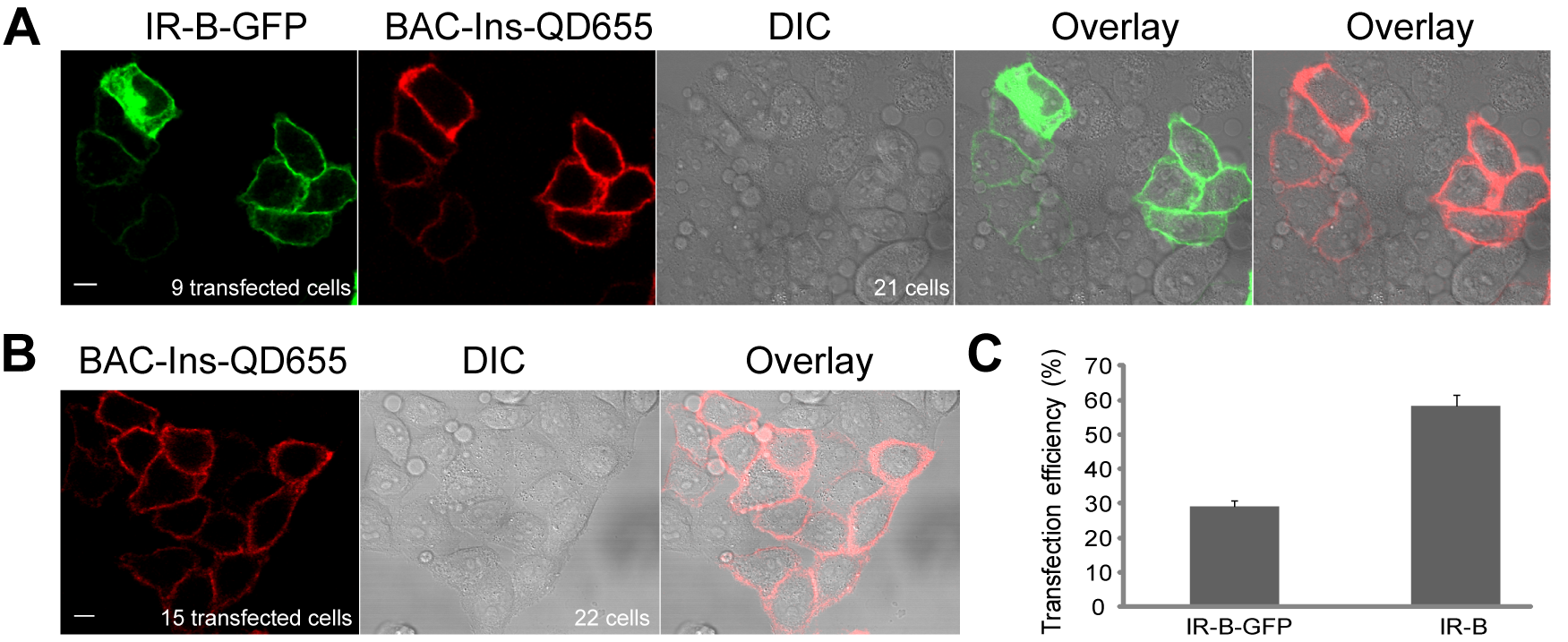

Supplement: Additional file 2: Figure S2 — Estimation of transfection efficiency. HeLa cells were transfected with pcDNA3-IR-B-GFP (A) or pcDNA3-IR-B (B) and labeled with 50 nM BAC-Ins for 15 min and 1nM QD655 for 10 min. After labeling cells were imaged by confocal microscopy (Zeiss LSM 510 Meta). Scale bars: 10 μm. C. Total number of cells was estimated from DIC images. Transfected cells were estimated from GFP images for IR-B-GFP and from QD655 images for IR-B. Results are expressed as the mean ± s.e.m (n = 219 cells for IR-VFP and n = 308 cells for IR from at least 3 images from 3 independent experiments). [file 1478-811X-11-18-S2.tiff]

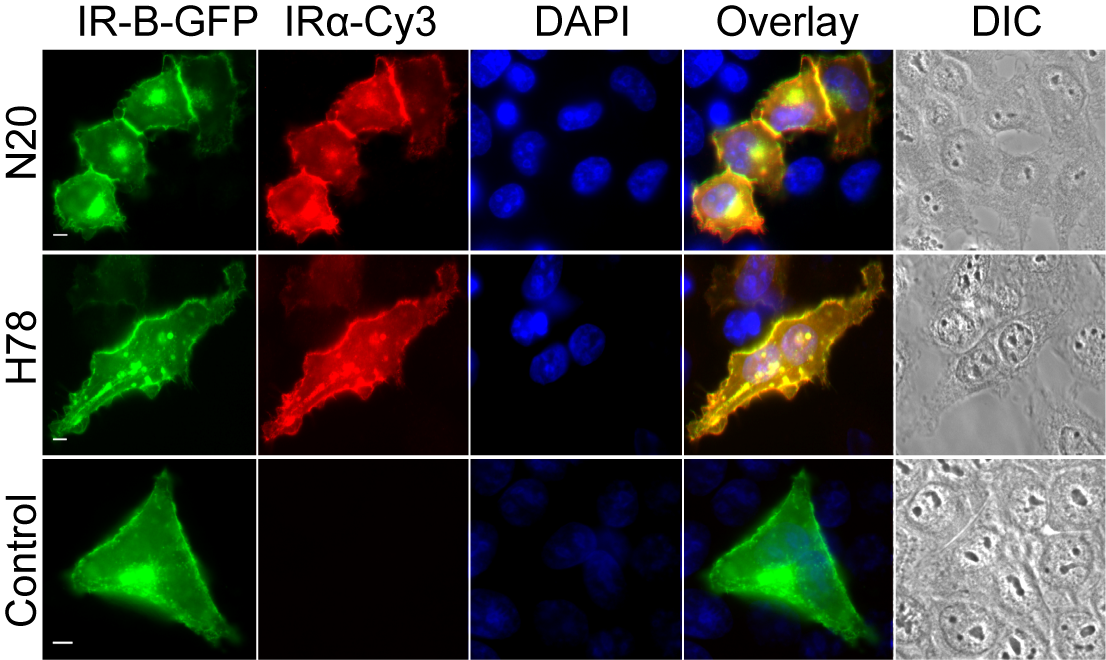

Supplement: Additional file 3: Figure S3 — Expression of IR-B-GFP by immunofluorescence. HeLa cells expressing IR-B-GFP were fixed in methanol and then incubated with primary antibodies against two different regions of the N-terminus of the α-subunit of IR (N20 and H78). Secondary antibody was conjugated with Cy3. Imaging was carried out by epifluorescence microscopy (Zeiss Axiovert S100). Scale bars: 5 μm. [file 1478-811X-11-18-S3.tiff]

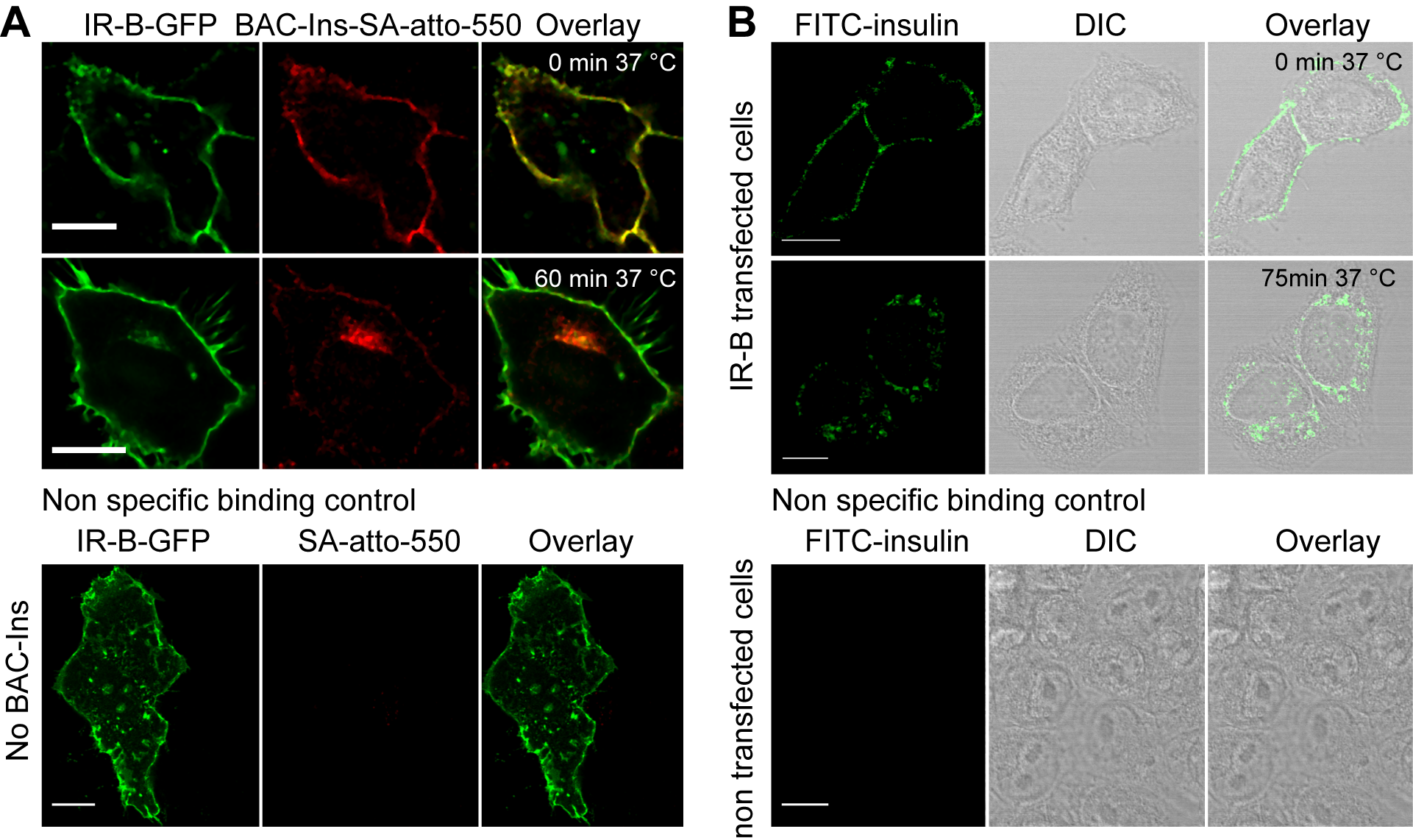

Supplement: Additional file 4: Figure S4 — Internalization of FITC-Insulin and BAC-Ins-SA-atto 550. A. HeLa cells over-expressing IR-B-GFP were labeled with 50 nM BAC-Ins for 15 min and then with 1 nM SA-atto 550 for 10 min at RT. Cells were directly fixed in PFA (upper panel) or incubated at 37°C for 60 min before fixation (middle panel). Lower panel shows the control experiment where the cells were treated similarly but without incubation with BAC-Ins. Imaging was performed by confocal microscopy (Olympus Fluoview FV1000). Scale bars: 10 μm. B. HeLa cells transfected with pcDNA3-IR-B were labeled with 50 nM FITC-insulin for 15 min and then directly fixed in methanol (upper panel) or incubated at 37°C for 75 min before fixation (middle panel). Lower panel shows similar experiment but in non transfected HeLa cells. Imaging was performed by confocal microscopy (Zeiss LSM 510 Meta). Scale bars: 10 μm. [file 1478-811X-11-18-S4.tiff]

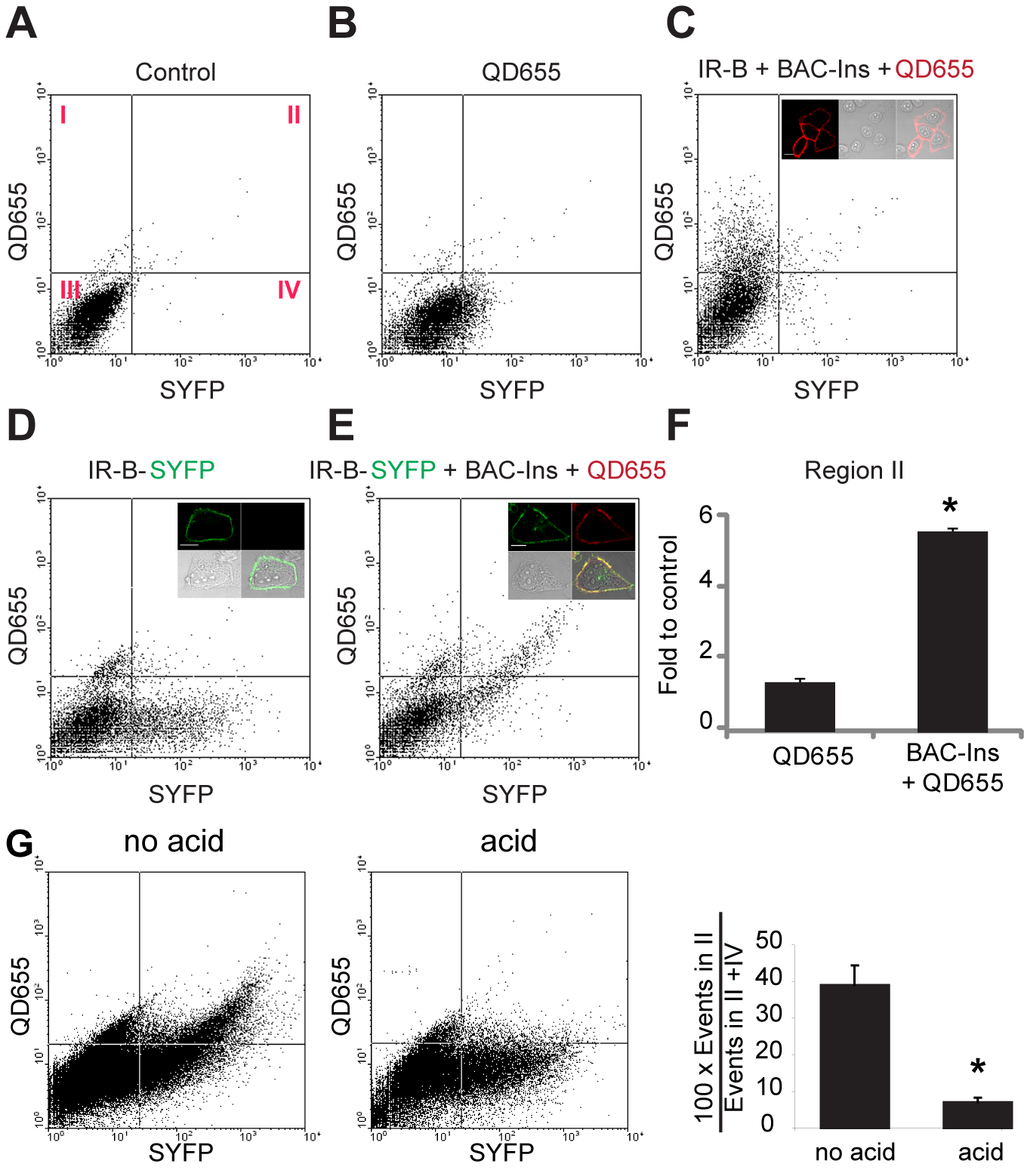

Supplement: Additional file 6: Figure S5 — Specificity of the binding of BAC-Ins and IGF-II-biot to the IR-B by flow cytometry. HeLa cells over-expressing IR-B (A-C) or IR-B-SYFP (D and E) were labeled in vivo with 50 nM BAC-Ins and 1 nM QD655 (C and E) or only with 1 nM QD655 (B and D). Cells were collected with 0.5 mM EDTA in PBS and were analyzed by flow cytometry detecting SYFP and QD655 signals. The images inside the graphs correspond to similar experiments but analyzed by confocal microscopy (QD655 are shown in red and SYFP in green). F. Quantification of the proportion of events in the region II (see panel A) with high signal of SYFP (transfected cells) and high signal of QD655 (insulin or IGFII binding). We normalized this value to the proportion obtained for the cells only incubated with QD655 without biotinylated ligand. G. HeLa cells expressing IR-B-SYFP were labeled in vivo with 50 nM IGF-II-biot and 1 nM QD655 at room temperature and were treated (or not) with acid (0.1 M Na-glycine pH 3, 0.5 M NaCl) for 2 min. After washing with PBS, cells were collected with 0.5 mM EDTA in PBS and analyzed by flow cytometry detecting SYFP and QD655 signals. The bar graph shows the quantification of the ratio between the events inside region II and the events inside region (II + IV). Asterisks indicate significant differences (p ≤ 0.001; n = 3 independent experiments). [file 1478-811X-11-18-S6.tiff]
