# Supplementary material for: Insulin and insulin like growth factor II endocytosis and signaling via insulin receptor B
Source: Cell Commun Signal. 2013 Mar 11;11:18. doi: 10.1186/1478-811X-11-18 (PMC3607927; doi:10.1186/1478-811X-11-18)
Supplement: Additional file 7 — Supplementary methods. [file 1478-811X-11-18-S7.doc]

**SUPPLEMENARY METHODS:**

**RT-PCR experiments.** cells were washed twice with PBS and RNA was extracted with Trizol according to manufacturer instructions. RNA was DNase treated using DNase I and reverse transcription was performed with High Capacity cDNA Reverse Transcription kit according to the respective protocols. PCR reactions were carried out with Gotaq DNA polymerase following manufacturer protocols. Primers for IR splicing analysis were: hIR-spl-F: 5´-tgaggattacctgcacaacg-3´ and hIR-spl-R: 5´- accgtcacattcccaacatc-3´. Product sizes were 120 nt and 84 nt for exon 11 inclusion and skipping respectively. Primers for mRNA levels were: hIR-F: 5´- aagtgcatccctgagtgtcc-3´ and hIR-R: 5´- tggtcttctcgccttctagg-3´; hIGF-IR-F: 5´- gactccgaggggtttgtgat-3´amd hIGF-IR-R: 5´- accttcacaagggatgcagt-3´; hGADPH-F: 5´- gattccacccatggcaaat-3´ and hGADPH-R: 5´- ctccatggtggtgaagacg-3´. Product sizes were: 115 nt for IR, 105 nt for IGF-IR and 170 nt for GADPH. All the primers were designed with similar parameters (GC content and melting temperature) using Primer3 software. Amplification program was: *i*) 1 min 15 sec at 95 °C, *ii*) 45 sec at 95 °C, *iii*) 45 sec at 57 °C, *iv*) 1 min at 72 °C , *v*) steps *i* to *iv* were repeated 25 times, *vi*) 10 min at 72 °C, *vii*) 5 min at 25 °C. PCR products were analyzed in 6 % polyacrylamide gels. Quantification was performed by densitometry by Image J plugins (gel analysis).

**Labeling *in vivo* with FITC-insulin.** Before the experiment cells over-expressing IR-B were starved overnight, washed with Tyrode’s buffer at room temperature and incubated with 50 nM FITC-Insulin for 15 min, washed with Tyrode’s and either fixed in methanol for 30 min at -20 °C or incubated at 37 ºC in DMEM for different periods of time before fixation.

**Labeling *in vivo* with IGF-II-biot or BAC-Ins and streptavidin-atto 555:** Labeling was performed as described in the main text using 1 nM streptavidin-atto 555.
